# Exploring monocarboxylate transporter inhibition for cancer treatment

**DOI:** 10.37349/etat.2024.00210

**Published:** 2024-02-23

**Authors:** Tomas Koltai, Larry Fliegel

**Affiliations:** University of Porto, Portugal; ^1^Hospital del Centro Gallego de Buenos Aires, Buenos Aires 2199, Argentina; ^2^Department of Biochemistry, Faculty of Medicine, University of Alberta, Edmonton T6G 2R3, Alberta, Canada

**Keywords:** Monocarboxylate transporters, glycolytic metabolism, lactate, lactate shuttle, quercetin, diclofenac, AZD3965

## Abstract

Cells are separated from the environment by a lipid bilayer membrane that is relatively impermeable to solutes. The transport of ions and small molecules across this membrane is an essential process in cell biology and metabolism. Monocarboxylate transporters (MCTs) belong to a vast family of solute carriers (SLCs) that facilitate the transport of certain hydrophylic small compounds through the bilipid cell membrane. The existence of 446 genes that code for SLCs is the best evidence of their importance. In-depth research on MCTs is quite recent and probably promoted by their role in cancer development and progression. Importantly, it has recently been realized that these transporters represent an interesting target for cancer treatment. The search for clinically useful monocarboxylate inhibitors is an even more recent field. There is limited pre-clinical and clinical experience with new inhibitors and their precise mechanism of action is still under investigation. What is common to all of them is the inhibition of lactate transport. This review discusses the structure and function of MCTs, their participation in cancer, and old and newly developed inhibitors. Some suggestions on how to improve their anticancer effects are also discussed.

## Introduction

Glycolytic metabolism and high glycolytic flux are hallmarks of cancer metabolism [1, 2]. This was established by the original research of Otto H. Warburg a hundred years ago [3, 4]. Most tumors, although not all, are highly dependent on glycolytic metabolism [5]. It has been shown that impeding glycolysis can decrease or even eliminate many of the characteristics of the malignant phenotype [6]. This is the reason why so much research has been devoted to curbing glycolysis. Different drugs have been tested in this endeavor. Many of them have shown very encouraging results, but no pharmaceutical agent has been found that impedes glycolysis ideally. Most of the pharmaceuticals, although useful at the experimental level, have not made their way to the bedside.

The final product of the glycolytic pathway is lactate. Lactate does not remain in the cytoplasm, where it is produced; it is swiftly extruded from the cell towards the extracellular compartment. If it was not exported and remained in the cytoplasm, intracellular lactic acidosis would rapidly ensue. This situation would jeopardize malignant cell survival. Therefore, this is a problem that cells have adapted to solve as quickly as possible. In situations where lactate export is totally blocked, there are three cellular responses that occur:

(A) Switch the glycolytic metabolism into a mitochondrial oxidative one. This results in the reduction or abolition of lactate production.
(B) Use the Cori cycle to regenerate pyruvate or glucose from lactate. This prevents lactate build up.
(C) Undergo apoptosis because of acidic stress.

Of options 1 and 2 above, option 1 may be preferred. The Cori cycle of pathway number 2 has limited usefulness because it uses high amounts of energy and quickly leads to energetic imbalance. The high amount of pyruvate formed also curbs further the Cori cycle function. Therefore, the more advantageous solution for cells is option 1 and the shift to oxidative metabolism.

In a malignant cell, however, a shift to oxidative metabolism has different problems, such as the chronic shortage of oxygen that usually occurs in the hypoxic environment of tumors. Additionally, in tumors, there tends to be no substrate for the generation of building blocks for non-essential amino acids, nucleic acids, and antioxidants. Oxidative metabolism produces a much greater amount of energy [36 adenosine triphosphates (ATPs)] than glycolysis (2 ATPs), but it is considerably slower, and rapid production of energy molecules requires glycolysis [7]. Thus, with the compound problems of relatively slow oxidative metabolism and impairment by lack of oxygen, a tumor cell deprived of the glycolytic pathway decreases or even stops proliferation and invasion.

**Figure 1:**
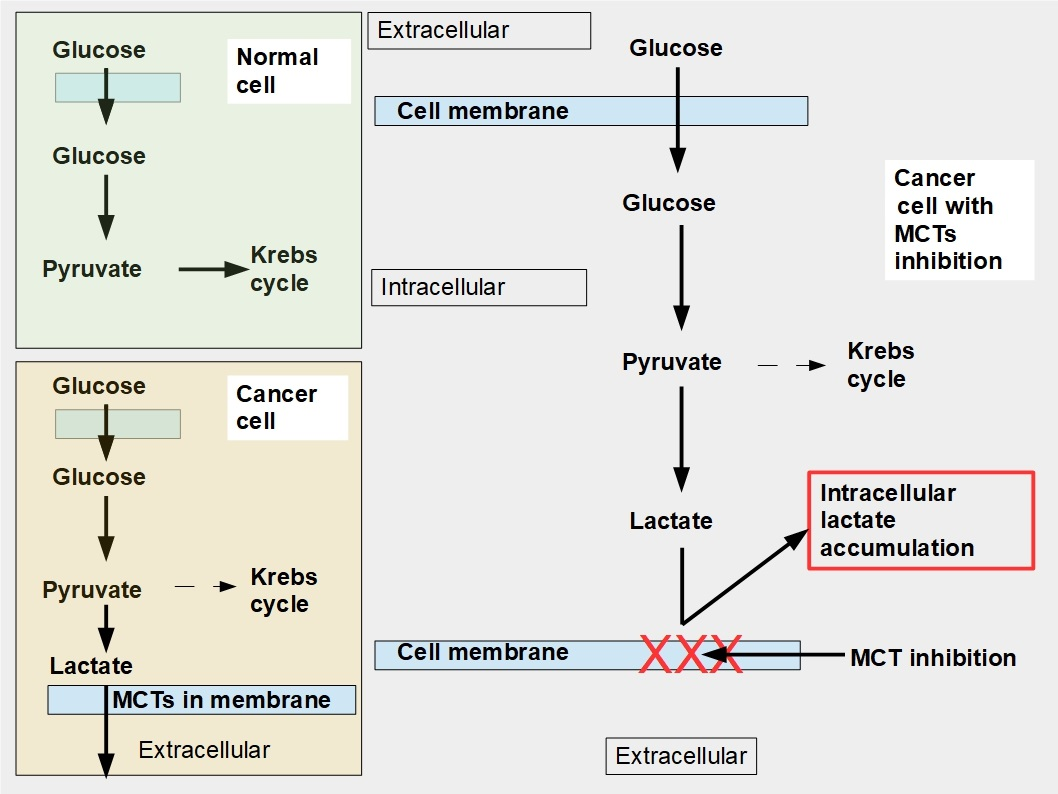
Glucose metabolism in normal and malignant cells and cells with monocarboxylate transporter (MCT) inhibition. Upper left panel: glucose metabolism of normal cells. All the pyruvate is metabolized through the Krebs cycle. Lower left panel: pyruvate is mainly catabolized to lactate, although a smaller proportion still follows the Krebs cycle pathway. Lactate is exported to the extracellular space by MCTs. Right panel: when MCTs are inhibited lactate cannot leave the cell and accumulates in the cytoplasm. In this situation, to avoid excessive and toxic lactate accumulation, the cell needs to revert to oxidative metabolism (not shown in the diagram). Normal cells can also adopt an anaerobic metabolism under hypoxic conditions. However normal cells return to oxidative metabolism as soon as hypoxia disappears (Pasteur effect), while cancer cells continue with mainly glycolytic metabolism (Warburg effect or aerobic glycolysis)

An alternative to the above 3 specific cell and metabolic approaches is to limit lactate extrusion. If lactate extrusion can be partially impeded, the cell would probably survive but intracellular pH would decrease, and likely the cell’s chance of proliferation would be curtailed. Blocking lactate extrusion is one way to cause a shift from a glycolytic to an oxidative metabolism [8]. That is the subject of this proposal (Figure 1), understanding and manipulation of mechanisms involved in lactate trafficking and how to inhibit it with pharmaceuticals. This chapter of onco-pharmacology has only recently become a very active field of research.

## Mechanism of lactate export/import

In mammalian cells, lactate is “transported” across plasma membranes by two types of transport facilitators:

(A) Proton-coupled MCTs that belong to a larger family of the solute carrier 16 (SLC16) [9]. This family of carriers has only four transporters that facilitate the movement of monocarboxylates, such as lactate, through membranes [10]. They are known as MCT1 to MCT4.
(B) Sodium-coupled MCTs or sodium MCT (SMCT, *SLC5A8* gene) [11]. Originally identified as a short fatty acid transporter, this is now also known to transport other monocarboxylates, including lactate [12].

MCTs are also facilitators of the transmembrane movement of other monocarboxylates such as pyruvate and ketone bodies. MCT1 and MCT4 are the main transporters of lactate. MCT1 can facilitate cellular lactate efflux and influx. When it exports lactate, it simultaneously increases intracellular pH because along with the lactate it facilitates proton extrusion in an equimolecular proportion. When a cell is submitted to anoxic conditions it switches to anaerobic glycolysis producing large amounts of lactate that are exported by MCT4 and MCT1. Cancer cells preferentially use glycolytic metabolism in a process called the Warburg effect whether they are hypoxic or not. Thus, cancer cells become dependent on MCTs to maintain their glycolytic metabolism. Without MCT export of lactate, intracellular lactic acidosis would inhibit cancer cell growth and survival.

In contrast to the MCTs, SMCT seems to have a tumor suppressor function [13], which apparently depends on its butyrate transport abilities [14].

## MCT1–4

**Figure 2:**
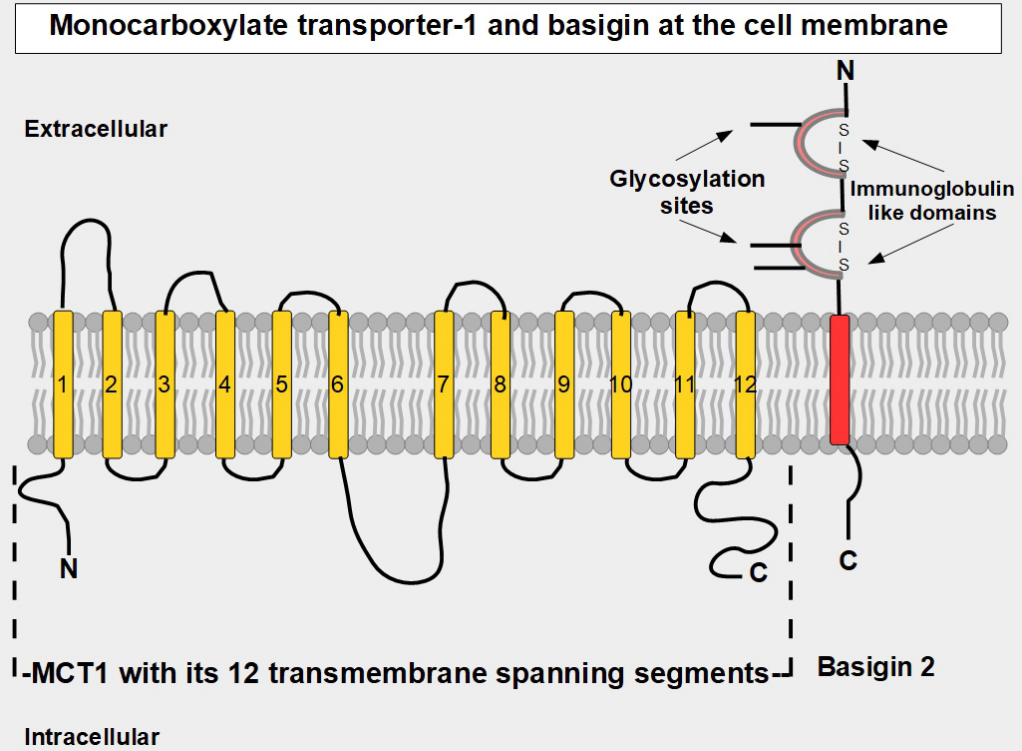
Linear two-dimensional model of MCT1 structure and basigin in the cell membrane [16–19]. MCT1 has 12 transmembrane segments, intracellular N-terminal and C-terminals, and a large intracellular loop between segments 6 and 7. Basigin (CD147 or EMMPRIN) although a separate protein, is functionally part of the transporter acting as a chaperone. MCT1–4 passively transport monocarboxylate ions and protons along the concentration gradient [20]. Basigin 2 has three glycosylation sites [21]. Interference with disulfide bridges inhibits basigin’s activity. S – S: disulfide bridge

**Figure 3:**
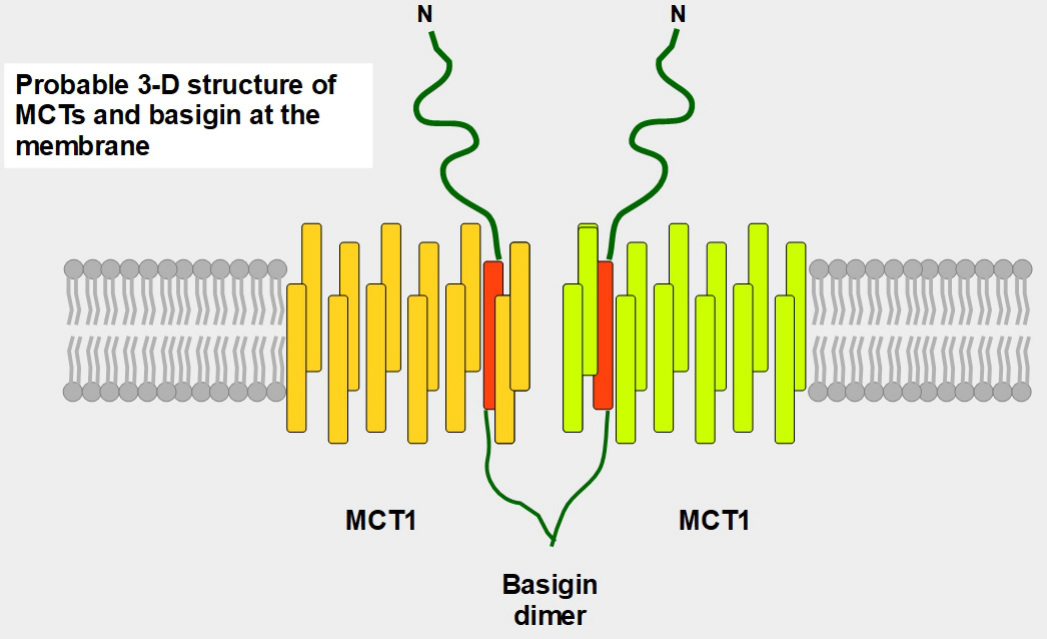
Postulated 3-D structure of the basigin dimer and two MCTs. The basigin dimer structure is based on references [22, 23], and modified from Wilson et al. [24]. Basigin dimerization depends on the extracellular domain [25], it occurs spontaneously *in vitro* [26] and probably influences the effects of the numerous basigin binding partners [27] such as cyclophilins, glucose transporter 1 (GLUT1), CD44, galectin 3, E-selectin among others. Importantly, basigin dimerization is essential for becoming fully functional [25]. Interestingly, basigin also binds the spike S protein of the severe acute respiratory syndrome-coronavirus disease (SARS-COVID) virus [28] and malarial parasites [29]. The intracellular and transmembrane domains are essential for MCT migration to the cell membrane [30]. The expression of basigin is essential for glycolytic tumor energetics [31–34]

MCT1–4 are the only bidirectional transporters of lactate. Therefore, this paper will focus exclusively on them. MCT1–4 are cell membrane proteins that span the membrane 12 times. For proper function, they require a tightly associated chaperone molecule that spans the membrane only once: basigin, also known as CD147 or extracellular matrix metalloproteinase inducer (EMMPRIN, Figures 2 and 3) [15]. A three-dimensional (3-D) view of MCT1 in the cell membrane is shown in Figure 3.

MCT1 contains 500 amino acids organized into 12 transmembranous segments that are united by extracellular and intracellular segments. MCT4 has 465 amino acids and a similar organization in the cell membrane. This organization allows MCTs to bundle in such a manner that a passageway is formed for the transported substrates [16]. They are ATP-independent. While MCT1 has a high affinity for lactate, MCT4 has a much lower one. MCT1 is an important lactate importer into oxidative cells and MCT4, although it is a bidirectional transporter, is better adapted for lactate export when intracellular lactate concentration is high. The lower affinity of MCT4 for lactate means that, though theoretically, it can import lactate into cells, practically speaking it rarely facilitates lactate import into tumor cells because the extracellular concentration of lactate would not be sufficient for binding and uptake. Similarly, MCT1 also exports lactate, and the knockdown of MCT1 inhibits tumor growth [17]. We believe that MCT1’s export ability is much higher than originally thought, as was shown by the intracellular lactate accumulation produced when the MCT1 inhibitor AZD3695 targeted cancer cells (see below).

## Basigin

Basigin is a cell surface protein of the immunoglobulin superfamily that has many other names such as EMMPRIN, which stands for extracellular matrix metalloproteinase inducer and CD147. It is encoded by the *BSG* gene in the short arm of chromosome 19. Basigin is strongly associated with the trafficking of MCTs to the cell surface [35] but has many other functions related to cancer. It functions as an inducer of matrix metalloproteases (MMPs), is a ligand of integrins, and functions in intercellular recognition, differentiation, and development. The name basigin comes from basic immunoglobulin [36].

Biswas et al. [37] cloned basigin and they identified an extracellular domain with two immunoglobulin subdomains, a transmembrane region with a short C-terminal portion. There are four splice variants.

Basigin is also a subunit of gamma-secretase complexes and down-modulates the production of beta-amyloid [38]. It participates in the process of invasion and its down-regulation decreases invasion [39]. Experiments to knockout or inhibit its activity have helped clarify its function. For example, when basigin null mice were created, MCTs were reduced in photoreceptors causing retinal degeneration [40]. Similarly, another group disrupted the basigin gene, and this strongly decreased the activity and expression of MCT1, MCT3, and MCT4 [41].

## Mechanism of function

**Figure 4:**
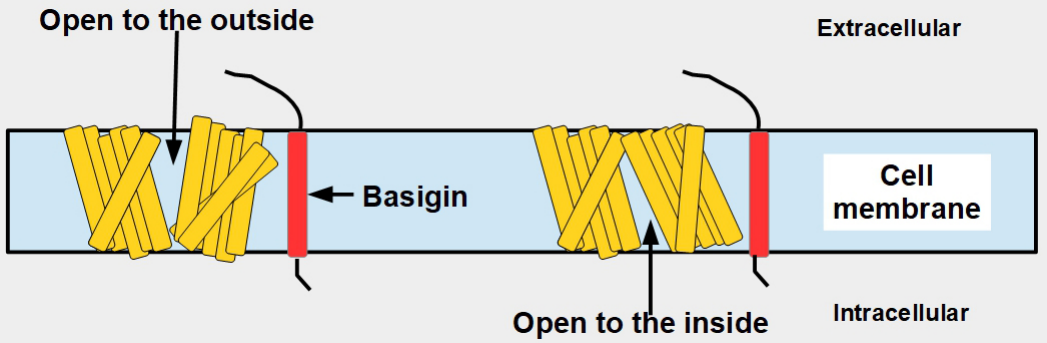
Conformational changes of MCT1 according to the direction of the transport

MCTs can extrude monocarboxylates in general and lactate in particular from the cell to the extracellular compartment, but they can also perform the opposite transport (particularly MCT1), that is, move monocarboxylates from the extracellular space into the cell. The mechanism by which this occurs was shown by Wang et al. [42], who demonstrated that the transporters undergo changes in conformation according to the direction of their transport (Figure 4). Their work reported four cryo-electron microscopy (cryo-EM) structures of MCT1/basigin complexes and demonstrated that substrate translocation is achieved through rigid-body rotation of the two domains which alternatingly expose the central substrate binding sites to either side of the membrane.

## Lactate shuttle

**Figure 5:**
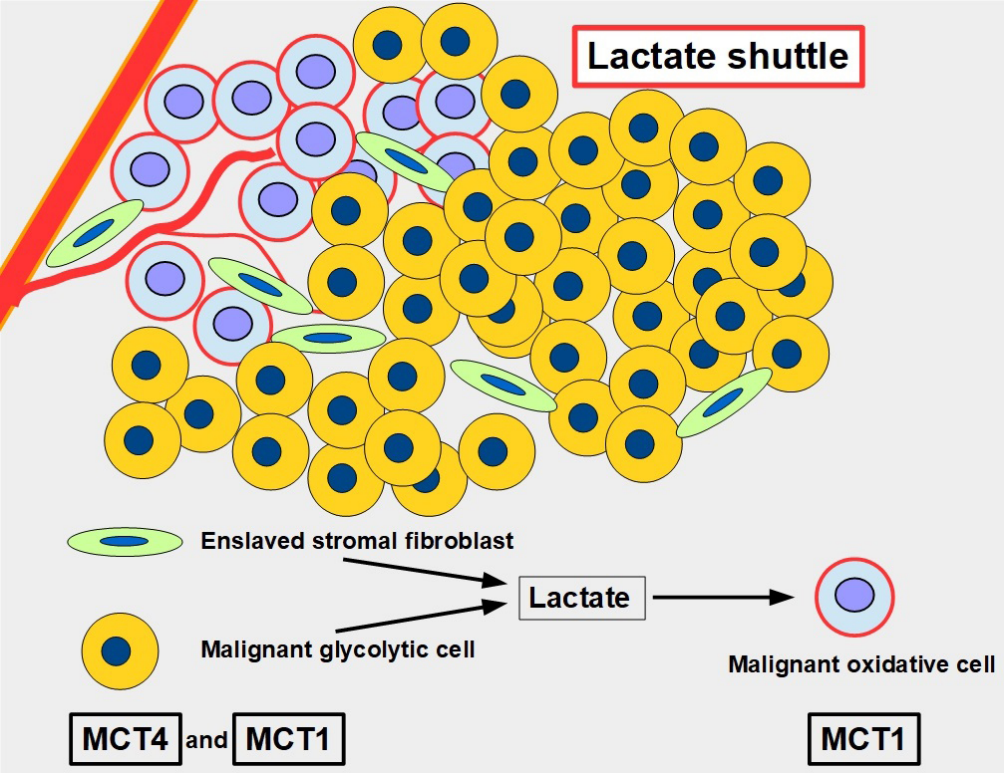
The lactate shuttle. Glycolytic malignant and glycolytic stromal fibroblasts produce and extrude lactate that is taken up by oxidative malignant cells near vascular supply. The lactate shuttle requires the primordial participation of MCTs. MCT4 is the main exporter of lactate in normal [53] and malignant cells, and MCT1 is the main importer

For many years, lactate was viewed as a metabolic end product, as a waste product. However, this concept was flawed [43]. Lactate is an energy source that is shuttled between cells [44]. The movement of lactate across different cell membranes requires the facilitator activity of MCT1 and MCT4. These shuttles work through a complex process that results in lactate being fed to oxidative cells. The lactate is initially produced in high amounts through glycolytic metabolism and the lactate transporters extrude this lactate into the extracellular matrix. From there it may be taken up by other more oxidative cells that use this lactate as a source of energy (Figure 5). Interestingly, tumor cells are not all glycolytic. There are other malignant cells, usually near well-oxygenated areas, that conserve an active and important oxidative metabolism. These cells can take up lactate and transform it into pyruvate feeding the Krebs cycle. Lactate therefore shuttles from glycolytic to oxidative cells. To have this circuit working, glycolytic cells need MCTs to extrude lactate, and oxidative cells also require MCTs to import lactate. Therefore, inhibiting the lactate transporters would block this shuttle which is an important mechanism promoting tumor growth [45]. This “lactate shuttle” is also found in normal cells [46, 47], but it acquires greater importance in cancer cells [48]. Furthermore, the lactate shuttle also works between stroma and tumor cells [49]. Some stroma cells are “enslaved” by the tumor which induces a metabolic change from oxidative to glycolytic metabolism. These cell-slaves extrude lactate to feed oxidative malignant cells [50, 51]. This phenomenon is known as the reverse Warburg effect (Figure 5) [52].

## MCTs and basigin in cancer

### MCTs

MCT1 and MCT4 are overexpressed in many cancers. MCT4 in particular, very efficiently transports lactate in environments with high lactate levels [54] and is well adapted to this transport in highly glycolytic cells [55]. Malignant cells use glycolytic metabolism even when there is sufficient oxygen for an oxidative metabolism (Warburg effect). This type of metabolism is frequently found not only in malignant cells but also in normal cells that are intensely proliferating. Evidently, this type of metabolism represents a growth advantage that took many years to be understood. Now, it is known that glycolytic metabolism allows intense proliferation in a medium depleted of oxygen. It reduces the generation of reactive oxygen species (ROS) and provides building blocks for the synthesis of macromolecules and molecules for antioxidant compounds. Furthermore, glycolytic metabolism can provide energy in a much faster way than oxidative metabolism, albeit at the cost of catabolizing ten times more glucose than usual oxidative metabolism for energy production [56–58]. The harsh and hypoxic environment in which cancers develop exerts a selective pressure on glycolytic cells, which are better adapted for survival [59, 60].

MCTs are over-expressed in many tumors, such as breast [61–63], colorectal [64–66], prostate [67–69], lung [70, 71], esophageal [72, 73], small cell lung [74], ovarian [75], gastric [76–78], endometrial [79], renal [80–82], hepatic [83, 84], pancreatic [85–87] cancers, leukemia [88, 89], lymphoma [90–92], head and neck squamous cell cancer [93–95], glioma [96], soft tissue sarcoma [97], and testicular germ cell tumors [98]. In summary, all kinds of tumors express MCT1–4, and in all of them, they play a role in malignant progression [99, 100]. Hypoxia is a strong driver for the increased expression of MTCs [101] and most tumors have large hypoxic areas.

A meta-analysis showed that MCT4 and its chaperone basigin (CD147) expression were correlated with poor clinical prognosis, including shorter disease-free survival and shorter overall survival across many different cancer types. There was no clear correlation with MCT1 [102]. This last finding does not have a clear explanation however fewer studies with high-quality data were available for this analysis.

### Basigin in cancer

**Figure 6:**
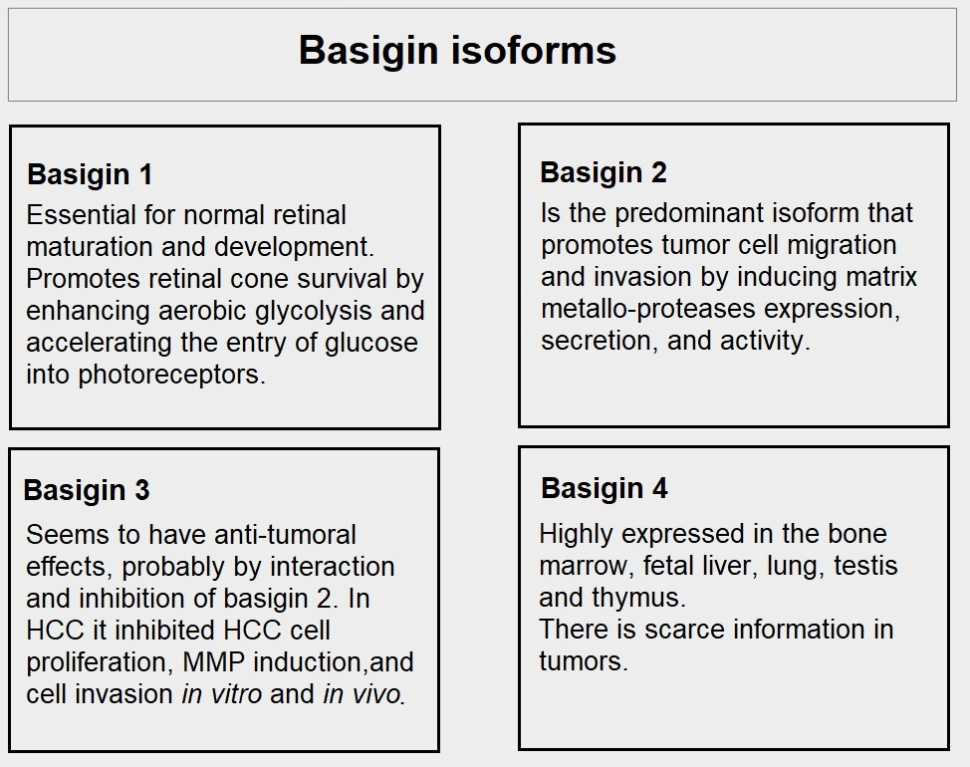
Differences among basigin isoforms. HCC: hepatocellular carcinoma

Basigin 2 (one of the four isoforms of basigin, see Figure 6) has been found to play an important role in the migration and invasion of ovarian carcinoma cells [103]. Similar findings occurred with melanoma cells [104]. Basigin knock-out blocked lactate export in non-small cell lung cancer (NSCLC) cell lines [105]. Basigin N-glycosylation by *N*-acetylglucosaminyl transferase V promotes metastasis in hepatocarcinoma [106]. MicroRNA Let-7-b is an endogenous suppressor of basigin expression, and it was shown to reduce invasion and metastasis in mouse melanoma cells [107].

## Mechanisms of tumor promotion by MCTs

The main mechanisms of tumor promotion by MCTs are exerted through lactic acid trafficking. The mechanisms are:

(A) The preservation of glycolytic metabolism by eliminating excess lactate.
(B) Raising intracellular pH to contribute to a hyperalkaline cytoplasm that favors proliferation.
(C) Maintaining the lactate shuttle which acts as a provider of lactate to non-glycolytic cells, thus contributing to energy balance (metabolic symbiosis between glycolytic and oxidative cells) [108].
(D) Contributing acid load to the acidic extracellular compartment which favors migration, invasion, and metastasis [109], contributing to the inversion of the pH gradient [110], and increasing angiogenesis through increased lactate uptake by endothelial cells [111], which occurs in several cell types including pancreatic ductal adenocarcinoma cells [85] and human lung cancer cells [112, 113].
(E) Low extracellular pH inhibits the immune response in tumors.

**Figure 7:**
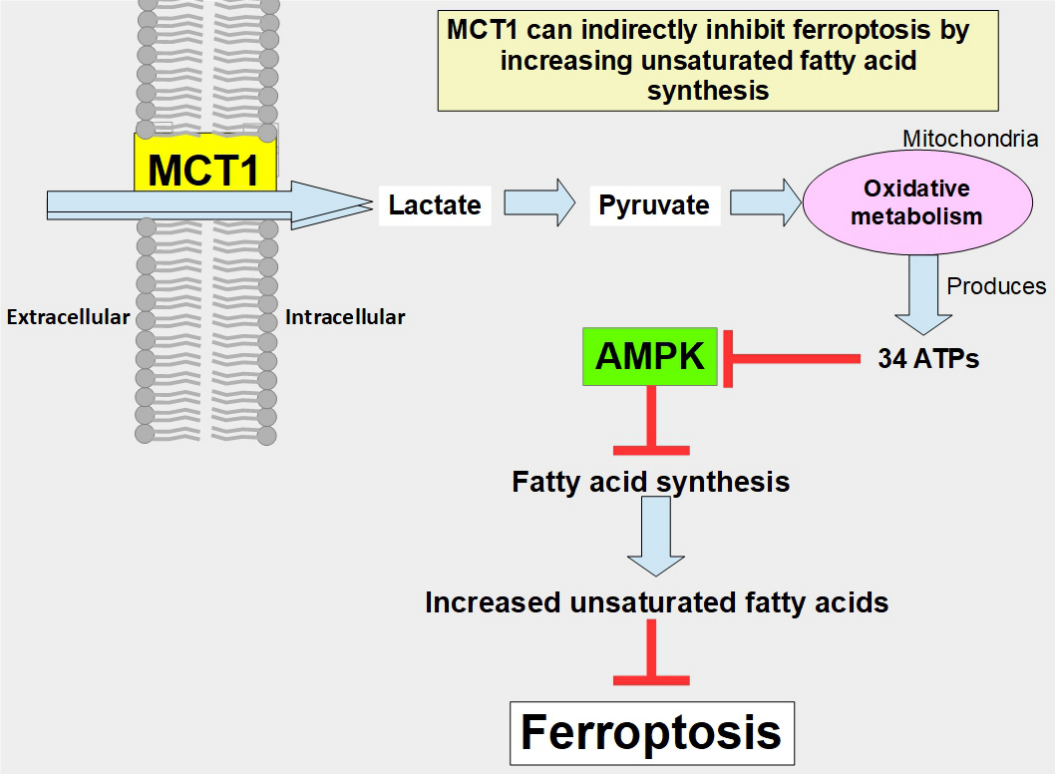
Pathway through which lactate uptake into the cell decreases sensitivity to ferroptosis. Lactate uptake promotes oxidative metabolism and ATP production. This increases the ATP/AMP ratio, thus downregulating AMPK activation. AMPK inhibition eliminates the restrain of fatty acid synthesis and unsaturated fatty acids which block ferroptosis

In addition to these central pro-tumoral activities, there are also other mechanisms in play that have been shown to act through MCT activity:

(A) MCT1 can indirectly prevent ferroptosis through the promotion of the production of anti-ferroptotic monounsaturated fatty acids. The mechanism is through the increased uptake of lactate which leads to increased ATP production, and through adenosine monophosphate-activated protein kinase (AMPK) inhibition that allows increased fatty acid synthesis. Proof of this concept was a study that showed that MCT1 inhibition increases ferroptotic susceptibility (Figure 7) [114]. Furthermore, the knockdown of MCT4 induced ferroptosis and inhibited autophagy in bladder cancer cells [115].
(B) Another study showed that inhibiting MCTs in lung cancer cells (A549) significantly decreased migration without affecting cell viability [116].

## MCT inhibitors

The pro-tumor activities of MCTs discussed above, suggest that MCT inhibition is a valid target for tumor treatment. MCT inhibition has been shown to reduce/inhibit the proliferative capability of malignant cells in vitro and importantly, *in vivo*, including in clinical studies in humans. Before considering each MCT inhibitor individually, a note of caution should be introduced: there are many drugs that can increase the expression of MCTs. This is the case with peroxisome proliferator-activated receptor α agonists such as clofibrate derivatives [117].

### Quercetin

**Figure 8:**
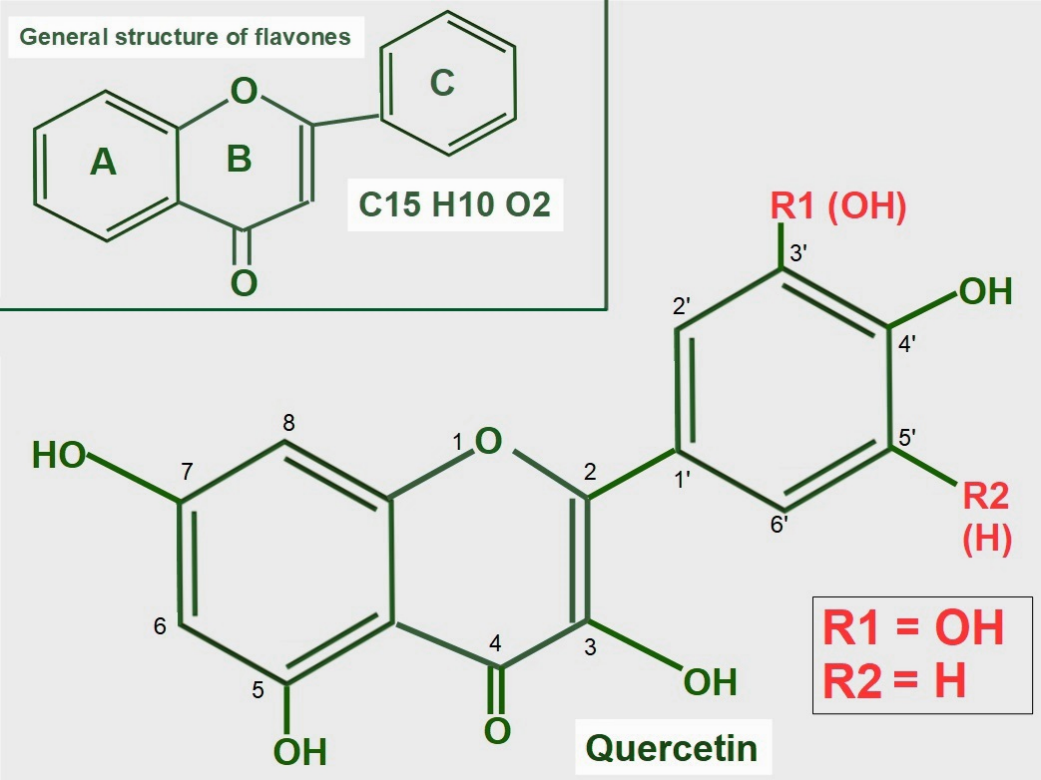
General structure of flavones and quercetin. Upper panel: flavonoids are polyphenolic compounds that have three rings: two phenyl rings (A and C) and a heterocyclic ring (B). Lower panel: quercetin’s formula (3,5,7,3’,4’pentahydroxyflavone) on the backbone of flavonols. Of note, it has five hydroxyl moieties

Quercetin is a flavonoid found in many foods in the usual human diet. Its widespread distribution in the vegetal kingdom may give the wrong impression that quercetin is only a nutritional supplement, which is how it is classified by the Food and Drug Administration (FDA). Quercetin is a flavonol, a subclass of flavonoids found in many plants, including fruits and vegetables as well as in seeds, nuts, and bark [118]. More than 5,000 flavonoids have been identified [119]. The normal human diet contains a small amount of quercetin, usually attached to a glycoside [120]. Flavonoids are a group of natural substances abundant in foods like fruits, plants, and some beverages. They have a three-ring polyphenolic structure in common: 2-phenylchromem-4-one (upper panel of Figure 8) [121]. Flavonoids can be modified to form many natural compounds, such as flavones, flavonols, isoflavones, and anthocyanins. Most authors also include chalcones in the group, despite it missing the B ring. The general structure of flavonols is shown in Figure 8.

Quercetin is a flavonol, one of the six subclasses of flavonoid compounds. Most flavonoids usually have glycosides (one or more sugars) attached. When they lack glycosides, they are known as aglycone (without sugars). The type of flavonol depends on the molecule attached in the R1 and R2 positions:

(A) Quercetin has an -OH at R1 and -H at R2 both in ring C.
(B) Kaempferol is another flavonol similar to quercetin, but lacks the -OH in the R1 position.
(C) Narigenin is a flavonol lacking a -OH in position 3 in ring B.
(D) Hesperetin shows a methylation in position 4’ of ring C.

Aglycones have very low water solubility, but they are soluble in lipids and alcohol. Adding sugar, whether glucose, rutinose, or rhamnose increases water solubility.

The structural formula of quercetin is shown in Figure 8. Most flavonoids have anti-tumoral effects, and they are all antioxidants. It is of note, that quercetin is the only one that has five hydroxyl groups, and this characteristic influences its effects.

Quercetin’s anticancer properties have been shown to act on different molecular targets and there is a great diversity of effects [122, 123]. These studies demonstrated preventive and therapeutic characteristics which will not be discussed here. For a review in this regard see Rauf et al. [124] and Ezzati et al. [125].

Various reports, including earlier studies, have characterized quercetin’s cellular activities. In 1969, Carpenedo et al. [126] discovered that quercetin inhibited mitochondrial ATPase activity. They concluded that quercetin “shows an affinity for membrane-dependent cellular activities”. The first papers showing that quercetin was able to rewire metabolism and inhibit glycolysis in malignant cells appeared in 1974 [127, 128]. The initial conclusion was that quercetin’s metabolic effects were due to inhibition of mitochondrial ATPase. In 1975, Suolinna et al. [129] worked with Ehrlich ascites tumor cells and discovered that hydroxyl-rich flavonoids (quercetin) can inhibit glycolysis. Furthermore, concentrations of quercetin between 5 μg/mL and 20 μg/mL induced growth inhibition. The explanation they gave was the “interfering with the generation of adenosine diphosphate and inorganic phosphate which are required for glycolysis”. However, it must be remembered that in 1975 the MCTs were not known. They also did not measure intracellular pH. Additionally, they found that ion pumps worked with greater efficiency. This is logical because now it is known that quercetin acidifies intracellular pH and the cells tried to survive by increasing the activity of sodium hydrogen exchanger 1 (NHE1) that exports protons. Therefore, unknowingly, Suolinna et al. [129] found that quercetin reduced the glycolytic behavior due to the accumulation of lactate.

Confirming our re-interpretation of the experiment, they found that the inhibitory effect of quercetin on glycolysis was hampered when the culture medium was enriched with bicarbonate. This is also logical, because the cell incorporates bicarbonate through sodium bicarbonate cotransporter 1 (NBC1), thus increasing intracellular pH. All these ion transporters were not identified at the time of the experiments.

Of all the flavonoids tested by Suolinna et al. [129], quercetin (at a concentration of 8 µg/mL) had the highest percent inhibition of lactate production (78%), meaning that it was the most efficient flavonoid to inhibit glycolysis. It was followed by luteolin with 75% inhibition (8 µg/mL) and kaempferol (16 µg/mL) with 52% inhibition. At a concentration of 40 µg/mL, quercetin completely inhibited lactate production. With 5 days of incubation, 5 µg/mL of quercetin reduced P388 leukemia cell growth to zero, while 2.5 µg/mL had no effect at all. Details of the mechanism of action of flavonoids were still missing at this time, though later, in 1979, Belt et al. [130] found that flavonoids, and in particular quercetin were potent inhibitors of lactate transport, and glycolysis, in Ehrlich ascites tumor cells.

More details were elucidated in the studies of Volk et al. [131] and Albatany et al. [132]. They induced intracellular acidification with quercetin through the inhibition of the MCT1 and MCT4 in glioma cells. This inhibition prevents lactate extrusion and increases intracellular lactate in malignant cells. Quercetin can inhibit lactate extrusion to the extent of increasing intracellular lactate levels 3-fold to 4-fold. At the same time, it decreases intracellular pH to 6.9. Importantly, Volk et al. [131] showed that these effects are limited to malignant cells. Unfortunately, the concentrations used to achieve lactate inhibition were in the order of 50 μmol/L which is higher than what can be achieved by oral administration. That does not mean that lower concentrations cannot achieve some positive results, but this possibility has not been fully tested [133].

Oral administration of quercetin can hardly achieve a level of 1 μmol/L in tissues, according to our study of the published literature. The highest concentration achieved was recorded by Graefe et al. [134]. Administering 100 mg oral quercetin to normal volunteers gave a peak plasma concentration of 2.3 μg/mL ± 1.5 μg/mL and a mean level of 2.1 μg/mL ± 1.6 μg/mL (approximately 7 μmol/L).

#### Quercetin phytosome and liposome

Quercetin phytosomes are phosphatidylcholine (or lecithin)-bound quercetin and quercetin liposomes are encapsulated quercetin. These forms of quercetin increase bioavailability many fold (quercetin and sunflower lecithin may be in a 1:1 weight ratio). According to the manufacturers, these formulations can increase quercetin absorption 20-fold [135, 136]. The highest concentration obtained after the ingestion of 500 mg of quercetin phytosome was 223 ng/mL (approximately 0.7 μmol/L) [135]. Unformulated quercetin absorption was only 11 ng/mL. Barras et al. [137] used a liposome loaded with quercetin that increased its solubility 100-fold. However, there is no information about the precise absorption levels.

#### Nanoparticles loaded with quercetin

There are no doubts that quercetin has a problematic biodynamics which casts doubts on its availability inside the cancer cell. Nanoparticles can solve this drawback, and much effort has been dedicated to this solution. Among the delivery systems assayed there are liposomes, silver and gold nanoparticles, poly lactic-co-glycolic acid (PLGA), polymeric micelles, nucleic acid conjugated micelles, and antibody-conjugated micelles [138].

Based on the data shown above we may theorize that 1 μmol/L to 7 μmol/L is probably the highest concentration achievable with oral or intravenous administration. Knowing that more than 90% of quercetin is bound to albumin, this leaves around 0.1 μmol/L to 0.7 μmol/L that can penetrate the tumor vicinity. Employing liposomes to deliver quercetin, its plasma concentration may improve 20-fold. Therefore, 2 μmol/L to 14 μmol/L is probably the maximum amount of quercetin that can be delivered to a tumor *in vivo* according to present technology. There are no publications regarding oral quercetin in doses higher than daily 500 mg. However, doses up to 1,000 mg can be used with no toxicity.

### Diclofenac

Many non-steroidal anti-inflammatory drugs (NSAIDs) have shown anti-tumoral effects such as decreased proliferation and growth, which was attributed to cyclooxygenase 2 (COX2) inhibitory abilities. In addition, non-COX2 dependent effects were found that modified glucose metabolism by decreasing GLUT1, lactate dehydrogenase A, and MCT1 expression in tumor cells [139].

**Figure 9:**
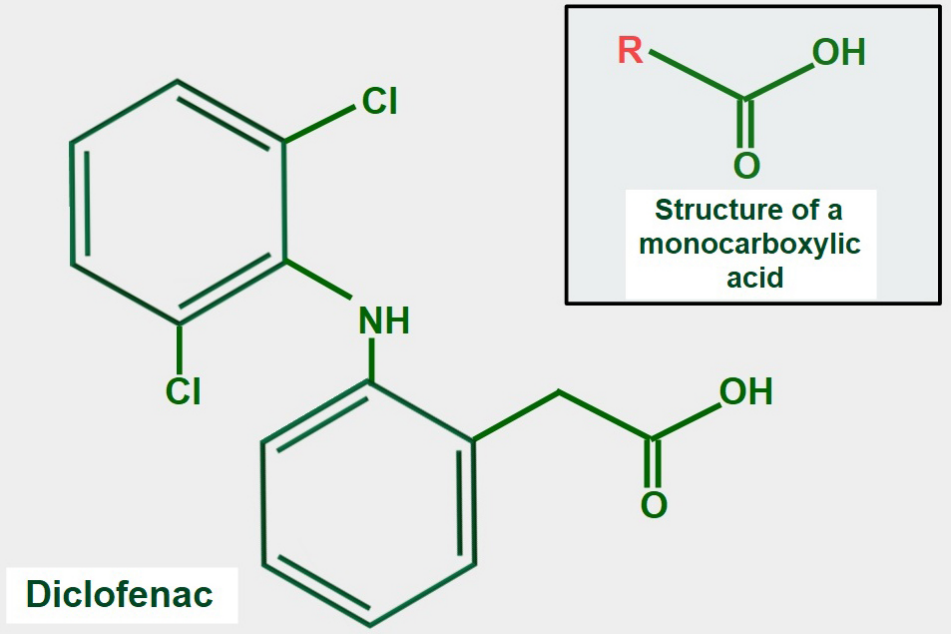
Chemical formula of diclofenac. The upper panel shows the general structure of monocarboxylate acids. Comparing both formulas, it is evident that diclofenac has a monocarboxylic acid group. This may explain the possible inhibitory effect on lactate transport by MCTs

Many NSAIDs have a monocarboxylic acid as part of their structure. It is attractive to speculate that NSAIDs may compete with lactate for MCTs, but this idea has not been fully confirmed experimentally. However, supportive evidence for the concept is that the NSAID diclofenac (Figure 9), interferes with the cellular uptake of gamma-hydroxybutyric acid, which enters the cell through the action of MCT1 [140]. Lactic acid and diclofenac also seem to compete in transplacental transport and may share the same transplacental transfer system [141]. There is additional evidence showing that MCTs play a role in monocarboxylic NSAID uptake [142]. A report by Sasaki et al. [143] showed that diclofenac exerted a powerful inhibitory effect on the lactate uptake of Caco-2 cells while Renner et al. [144] have shown that diclofenac can inhibit MCTs.

Interestingly, diclofenac has the opposite effect in retinal cells HRPE and ARPE-19 where it increases proton-coupled and sodium-coupled MCT activity [145]. This may be related to the high expression of basigin 1 in retinal cells, although there is no experimental evidence to sustain this idea.

### Syrosingopine

**Figure 10:**
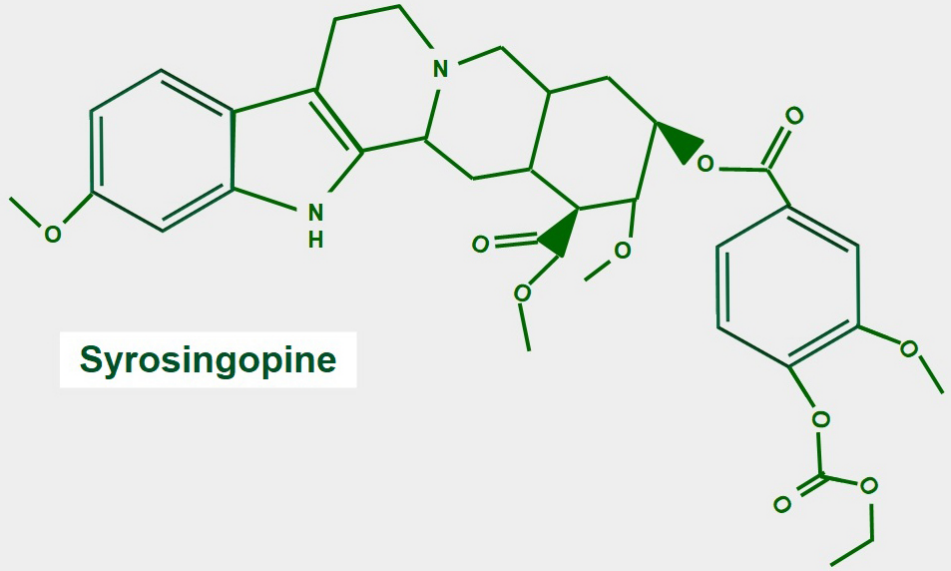
Chemical structure of syrosingopine. There are no studies regarding the mechanism of MCT inhibition

Syrosingopine (Figure 10) is a reserpine derivative used for treating hypertension. This is a weak antihypertensive medication that was developed by CIBA Pharmaceuticals (now Novartis) and first used in 1958. Due to poor sales, it was discontinued in 1968. Importantly, it is also a dual MCT inhibitor that targets MCT1 and MCT4. Evidence for its physiological actions comes from several studies. In a study by Buyse et al. [146], syrosingopine decreased extracellular acidity, glucose consumption, and lactate secretion, and importantly decreased tumor cell proliferation *in vitro*. However, it showed no effects *in vivo*. In contrast, in a different study at least at the cellular level, the co-administration of syrosingopine with metformin showed important anti-tumor effects. However, this work did not test the compound *in vivo*. Similarly, Benjamin et al. [147] found that syrosingopine sensitized cancer cells to the administration of far lower doses of metformin or phenformin than were necessary for anti-cancer effects with metformin or phenformin alone. We think that what happens is something complex with many contributing factors. Metformin or phenformin increases the production of lactate by inhibiting complex I in the electron transport chain. This additional load of lactate is added to that produced by the increased glycolytic metabolism, plus the load from the inhibition of its extrusion by syrosingopine. This leads the cell to acidic stress and eventual apoptosis. Supporting this theory is that the only confirmed effect of metformin in normal or tumor cells is inhibition of complex I. All the supposed antitumoral effects are secondary to this inhibition. Several authors also suggest the main effect of syrosingopine is due to intracellular acidification [148–150]. In another later publication, Benjamin [151] suggested that metformin potentiates syrosingopine through intracellular acidic stress-mediated effects.

### Statins

Statins have shown an ability to inhibit lactic efflux from muscle cells. In this regard atorvastatin seemed the most potent: it can cause a 2.5-fold increase in intracellular lactate. Simvastatin and rosuvastatin had no effects [152]. Regarding cancer cells, similar findings were observed with atorvastatin earlier, as were much lower effects of fluvastatin. Lovastatin showed no inhibitory effects [153]. Kobayashi et al. [154] found that lipophilic statins inhibited MCT4. On the other hand, hydrophilic statins had no such effects. In a recent publication, all statins were found to exert inhibitory effects on MCT1, MCT2, and MCT4. Atorvastatin showed selective and potent inhibition of MCT2 [155]. This is not an unimportant observation, because MCT2 is a high affinity pyruvate transporter [156].

Statin treatments are known to cause myotoxicity, and this is fully explained by their inhibition of MCTs [157]. However, in clinical practice, statin-related myotoxicity is a multifactorial development in which many other facts play a role, such as dose, concentration, interaction with fibrates, statin-induced interruption of glycoprotein synthesis in the muscle membrane, increased intracellular calcium concentrations leading to impaired membrane function and decreased membrane fluidity.

α-cyano-4-hydroxycinnamate, an MCT4 inhibitor also produced the same type of lesions induced by atorvastatin in embrional rabdomyosarcoma cells [158]. This seems bad news for the systematic use of statins for lowering cholesterol, but it is the opposite regarding their clinical utility in cancer.

Statins can achieve inhibition of MCTs. Atorvastatin, the most potent in this regard, was found to be a non-competitive inhibitor of MCT1 with a constant concentration of 40 μmol/L [159]. The problem is that the maximum achievable concentration of atorvastatin after a normal high dose does not go beyond nmol/L levels [160]. The unanswered question that remains here is: can clinically acceptable doses of atorvastatin inhibit MCTs? One study improved statin delivery to tumors by using cell-derived microparticules loaded with fluvastatin. These attenuated lung adenocarcinoma cells growth *in vivo* [161]. Improving pharmaceutical delivery methods through nanoparticles may be a useful avenue to pursue to increase the amount of statins deliverable to the tumor.

### Lonidamine

**Figure 11:**
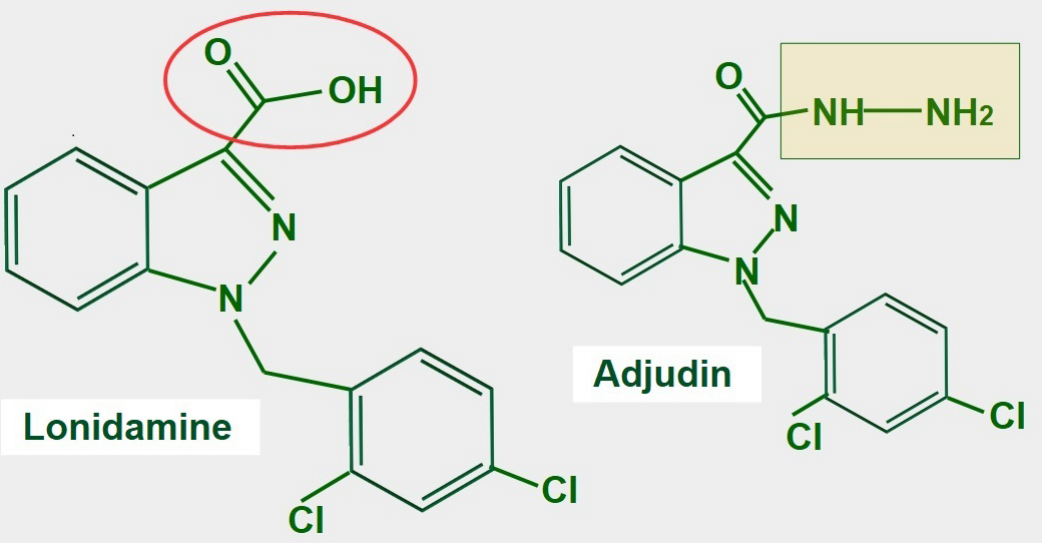
Chemical structure of lonidamine. The carboxylate group is indicated in red. The right panel shows the chemical structure of adjudin, a derivative of lonidamine

Lonidamine is a derivative of indazole-3-carboxylic acid (Figure 11). It is an old and almost forgotten drug with low toxicity, that interferes with glucose metabolism in different ways. Research is now in a period of rediscovery of what lonidamine can do in cancer cells and how it inhibits glycolysis.

Lonidamine was originally developed in the 1970s as a spermicide. Discovery of its anti-tumoral effects came shortly after [162, 163]. After more than 30 years of research, the exact mechanisms of action of lonidamine are not fully known. Lonidamine inhibits glycolytic and oxidative metabolism of glucose, decreasing the production of ATP whether mitochondrial or glycolytic. Furthermore, it also inhibits lactate extrusion, thus producing an important intracellular acidification.

**Figure 12:**
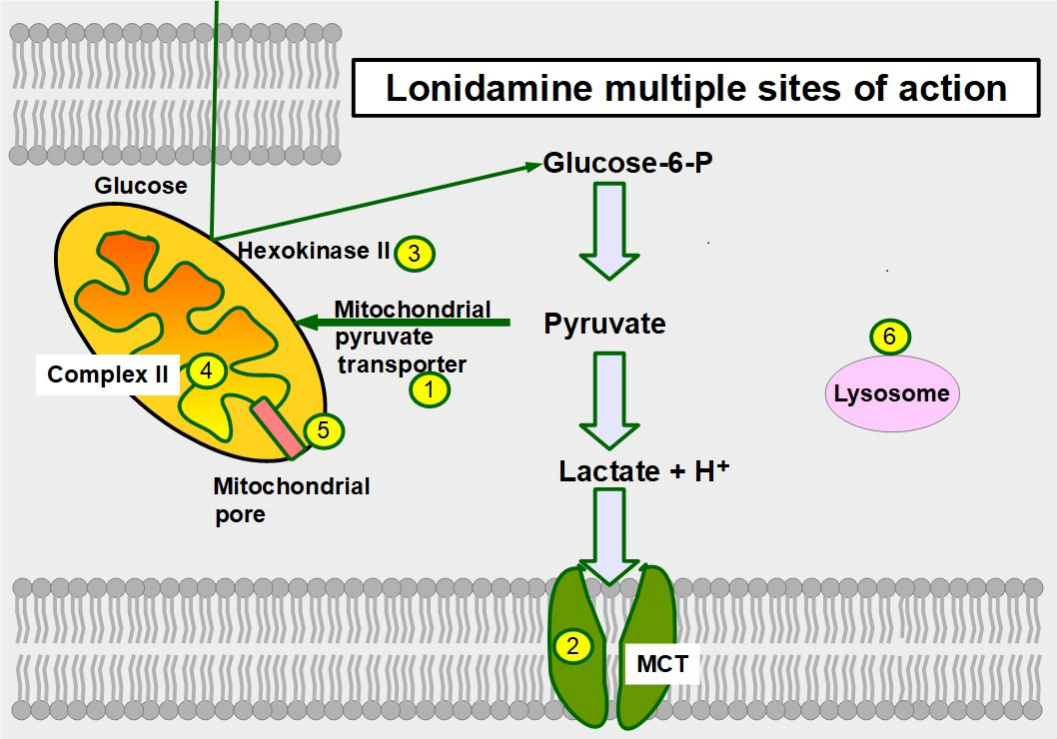
Sites of action of lonidamine in glucose metabolism. 1: inhibition of the mitochondrial pyruvate transporter; 2: inhibition of MCTs; 3: hexokinase II inhibition; 4: inhibiting complex II in the electron transport chain; 5: promoting opening of the mitochondrial permeability pore; and 6: lonidamine also acts on lysosomes interfering with their acidification

Six mechanisms of action have been identified (Figure 12):

(A) Inhibition of the mitochondrial pyruvate carrier which impedes mitochondrial uptake of pyruvate [164].
(B) Inhibition of MCT1, MCT2, and MCT4 thereby increasing intracellular lactate concentration and decreasing intracellular pH [165].
(C) Inhibition of complex I/II in the electron transport chain [166].
(D) Inhibition of hexokinase II, a driver of the glycolytic flux [167, 168]. Hexokinase II is particularly over-expressed in glycolytic cancer cells [169].
(E) Decreasing mitochondrial membrane potential, increasing mitochondrial membrane permeabilization [170], and inducing apoptosis, However, at clinically acceptable doses it is a weak inductor of apoptosis.
(F) Causing an activation (opening) of the mitochondrial permeability pore [171].

The main effect of lonidamine seems to be the targeting of the mitochondrial pyruvate transporter which it accomplishes with low concentrations. For MCT inhibition, much higher concentrations are required (above 150 μmol/L) [172]. However, all the effects mentioned above produce an important acidification of the cytoplasm and an energy restriction [173, 174]. Adjudin, a derivative of lonidamine showed similar antitumor effects [175].

The lack of positive results in cancer treatment with lonidamine is produced by its use as a stand-alone drug, which is not how it should be utilized. Its minimal toxicity permits it to be added to more toxic treatments without adding adverse effects, which may be a more productive use of the compound. To date, the clinical experience with lonidamine as a stand-alone drug has been poor. This may be because the drug has reversible, and short-duration effects and as noted above, because the drug was not administered with other anticancer drugs. When lonidamine was co-administered with other anticancer drugs, it was shown to potentiate their effects [176, 177]. In one example, when administered with metformin, lonidamine showed increased apoptotic effects *in vitro* and *in vivo* [178]. Pharmokinetic studies have demonstrated that lonidamine administered to humans can achieve concentrations high enough to block mitochondrial pyruvate transporter and MCTs [179–181].

The drugs described above are weak MCT inhibitors and usually require high concentrations to achieve inhibition. On the other hand, the drugs that will be considered below are direct MCT blockers and achieve inhibition at single-digit nanomolar concentrations, therefore they are much more potent than most of the above-named compounds.

### Cyano cinnamic acid derivatives

**Figure 13:**
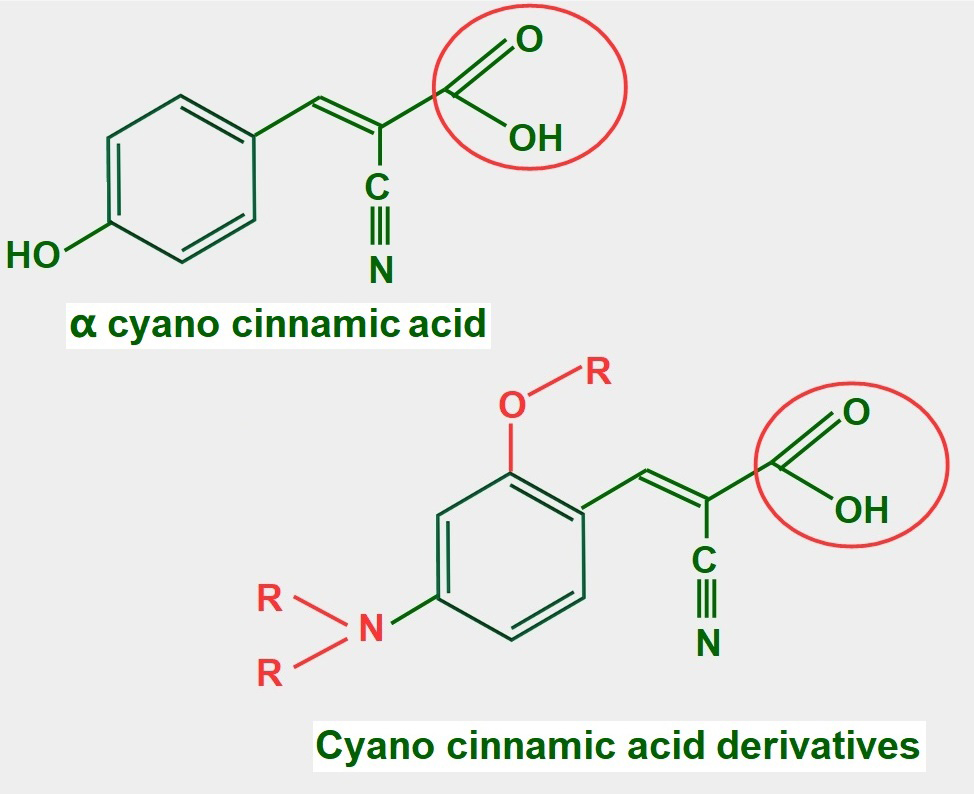
Chemical structure of cyano cinnamic acid and its derivatives. Note that they all have a conserved carboxylate (circled with a red line)

Cyano cinnamic acid (Figure 13) is the structural base from which two powerful MCT1 and MCT4 inhibitors have been developed [182]. Although the exact mechanism by which they inhibit MCTs is unclear, it was found that one of the derivatives, α-cyano-4-hydroxycinnamate bound strongly to the MCT without being translocated. However, it could be displaced by the addition of lactate [183].

Many of these derivatives have shown antitumoral effects *in vivo* [184]. They are still undergoing pre-clinical experimentation.

### BAY-8002

**Figure 14:**
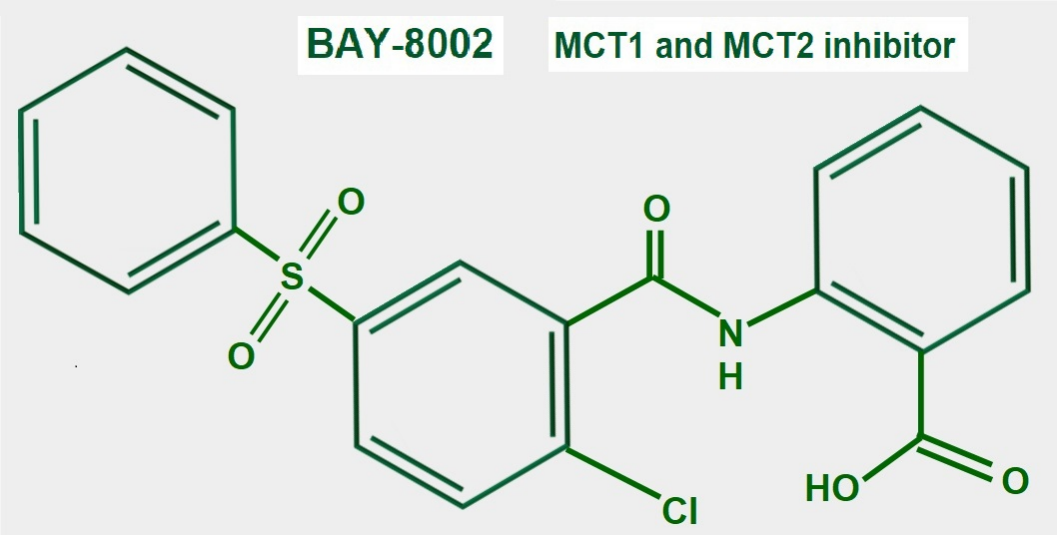
Chemical structure of BAY-8002

BAY-8002 (Figure 14) is an orally available potent dual MCT1/2 inhibitor that suppresses bidirectional lactate transport. Laboratory and pre-clinical studies showed that large B cell lymphoma cells were particularly sensitive to MCT1 inhibition by BAY-8002 (these cells lack MCT4 expression). BAY-8002 also increased intracellular lactate. Resistant cells developed increased MCT4 expression [185].

### 7-aminocarboxycoumarin 2

**Figure 15:**
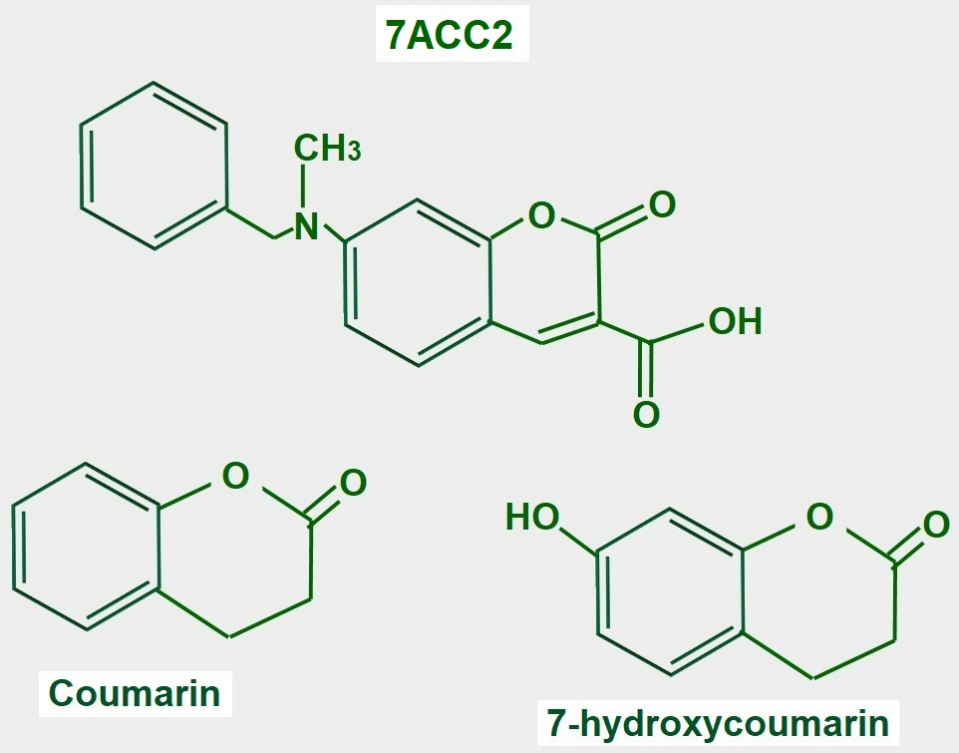
Chemical structure of coumarin and derivatives. The upper panel shows the chemical structure of 7ACC2. The lower panel shows the structure of the compound from where it is derived

7-aminocarboxycoumarin 2 (7ACC2, Figure 15) is a potent inhibitor of lactate influx into the cell. It has minimal or no effects on efflux, thus it is an inhibitor of MCT1 but not of MCT4. However, the laboratories that sell the product suggest that it inhibits both proteins (Merck KGaA, Darmstad, Germany).

7ACC2 was tested on cervical carcinoma (SiHa cell) tumors, colorectal HCT116 tumors, and xenografted orthotopic MCF-7 breast tumors, and delayed growth in all of them [186].

Several studies support the potential use of 7ACC2 in cancer treatment. Corbet et al. [187] mention the possibility that inhibiting lactate uptake via MCT inhibitors may increase glucose utilization in oxidative malignant cells. Importantly, they found that 7ACC2 also inhibited mitochondrial pyruvate transport. This leads to intracellular pyruvate accumulation which inhibits lactate uptake. They also found that 7ACC2 had an important radiosensitizing effect. Sandforth et al. [188] also showed that 7ACC2 reduced stemness in pancreatic cancer cells expressing MCT1. Unfortunately, its multiple off-target effects interrupted further development.

### AZD3965

**Figure 16:**
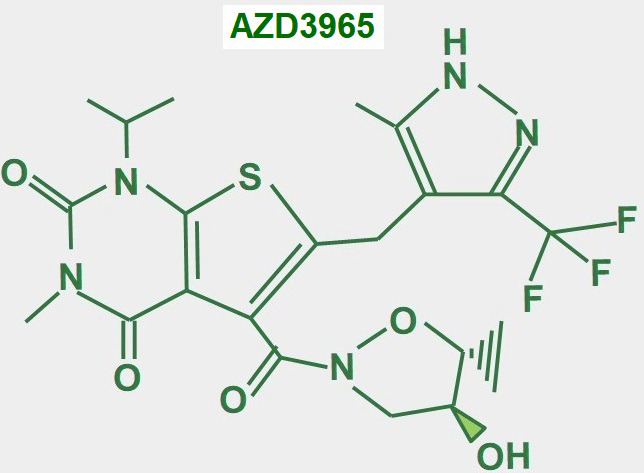
Chemical structure of AZD3965

AZD3965 (Figure 16) is an updated variant of AR-C155858, its predecessor, which was an MCT1/2 inhibitor [189]. AZD3965 is probably the most promising MCT1 inhibitor for cancer treatment. Some clinical trials have already been completed. There was a phase I clinical trial concluded with 35 patients with solid tumors. The 10 mg twice-a-day dose was shown to be the safest. Some adverse effects on the heart (troponin elevation) and retina (reversible electroretinographic changes) were also suggested [190]. In a phase I expansion study with the drug, a twice-daily dose of 10 mg was found to be safe. Of the 11 patients with diffuse large B cell lymphoma, 1 patient had a complete remission and 1 achieved stable disease [191]. A multicenter phase I trial of dose escalation in 40 patients with advanced solid tumors (mainly colorectal adenocarcinoma and mesothelioma) or lymphoma and no standard therapy options showed similar adverse effects on the retina and heart and grade 3 acidosis. The dose of 10 mg twice a day was confirmed as the most convenient. From 39 evaluable patients, 9 achieved stable disease as the best response [192].

*In vitro*, other studies have suggested that AZD3965 is more useful in tumors with high MCT1 and low MCT4 expression [193]. This may be explained by other results that showed that elevated MCT4 expression is a mechanism of resistance to MCT1 and MCT2 inhibition [194, 195].

In conclusion, the drugs’ utility is restricted to tumors with high MCT1 and low MCT4 expression. As a stand-alone drug, the performance of AZD3965 is poor.

According to the preliminary results with AZD3695, it is evident that some further co-administered drug is needed if a better outcome is desired. It may be possible to improve the effect of AZD3965 by co-administration with simvastatin as this showed additive effects in tumor growth delay in mouse-bearing head and neck squamous cell carcinoma (HNSCC) xenografts [196]. Metformin deserves to be tested in this regard. In 2016, one of us (Koltai T) proposed the double approach of increasing intracellular lactate production by inhibiting complex I in the electron transport chain, with metformin and simultaneously inhibiting lactate extrusion [197]. This theoretical idea was further developed by other authors [198] and was proposed by Benjamin and Hall [199] to be used with AZD3965 acting as a lactic acid extrusion inhibitor. By itself, to have a significant effect AZD3965 may require doses that are toxic, and results are poor. Co-administered metformin may allow the use of a lower dose of AZD3965 and may achieve higher toxicity in cancer glycolytic cells. However, it will likely have no effect on cancer oxidative cells.

### CYT-851

CYT-851 is an inhibitor of RAD51, the protein that participates in homologous recombination repair of DNA. It was discovered that CYT-851 is also an MCT inhibitor. It has been tested in phase I clinical trials in combination with capecitabine or gemcitabine [200]. In a cohort of 8 patients with heavily pre-treated solid tumors receiving capecitabine, there was 1 partial response and 7 cases of stable disease. In 6 patients of the gemcitabine cohort, there was one partial response, and 4 achieved stable disease (ClinicalTrials.gov identifier: NCT03997968). This drug also showed important activity in hematological tumors such as non-Hodgkin lymphoma [201]. Unfortunately, the company developing the drug has stopped operations due to financial problems.

## Basigin inhibitors

Inhibition of basigin with anti-sense RNA reduced invasion and angiogenesis in glioblastoma cells. It reduced MMP2, MMP9, and vascular endothelial growth factor (VEGF) [202].

There are only a few identified inhibitors of basigin:

(A) p-chloromercuribenzene sulfonate is an organomercurial that interferes with the disulfide bridges (Figure 2) and was used to study the protein *in vitro* [203].
(B) Acriflavine inhibits the binding between basigin and MCT4. Acriflavine treatment significantly inhibited grown and self-renewal of glioblastoma neurosphere lines in vitro, and treatment in mouse xenografts inhibited tumor progression [204]. Acriflavine is a topical antibacterial agent that has been found to be a potent inhibitor of hypoxia-inducible factor-1 (HIF-1) [205].

## Discussion

MCT1–4 are housekeeping transporters, and their full inhibition should have considerable toxicity. Something similar occurs with basigin. A partial inhibition, although toxic, is undoubtedly better tolerated. The main anti-cancer mechanism of MCT inhibition seems to be impeding lactate trafficking. All the deleterious effects on the tumor are a consequence of this interference with lactate movements. Regarding this movement, two alterations need to be distinguished with different effects:

(A) Lactate that cannot leave the glycolytic cell.
(B) Lactate that cannot penetrate malignant oxidative cells.

Lactate that cannot leave the glycolytic cell will have three effects:

(A) Intracellular lactate accumulation.
(B) Reducing intracellular pH causing intracellular lactic acidosis which hinders proliferation.
(C) Accumulation of the glycolytic product lactate which will decrease the upstream metabolism of precursors, such as glucose, that is, it will slow down glycolysis or even fully impede it.

Lactate that cannot penetrate oxidative cells will decrease energy substrates for the cell and reduce the ATP pool, thus impeding proliferation, and activating AMPK which in turn favors autophagy and apoptosis and inhibits lipid and protein synthesis.

The anti-tumoral dose of the potent MCT1 inhibitor AZD3965 did not show striking results as a stand-alone drug, and in addition, eye and heart toxicity was significant. To this, we must add that this drug does not inhibit MCT4 which can replace MCT1 and MCT2 and create an escape mechanism allowing tumor growth.

We believe, that AZD3965 may be a very useful drug as part of a different scheme that would allow for a much lower dose and toxicity. For example, if cell acidifiers were co-administered with AZD3965 it is highly possible that better results may be obtained. AZD3965 decreases intracellular pH by causing lactate accumulation but activates the NHE1 which then tries to compensate for the lower pH [206]. Inhibiting NHE1 should therefore increase AZD3965 efficiency.

**Figure 17:**
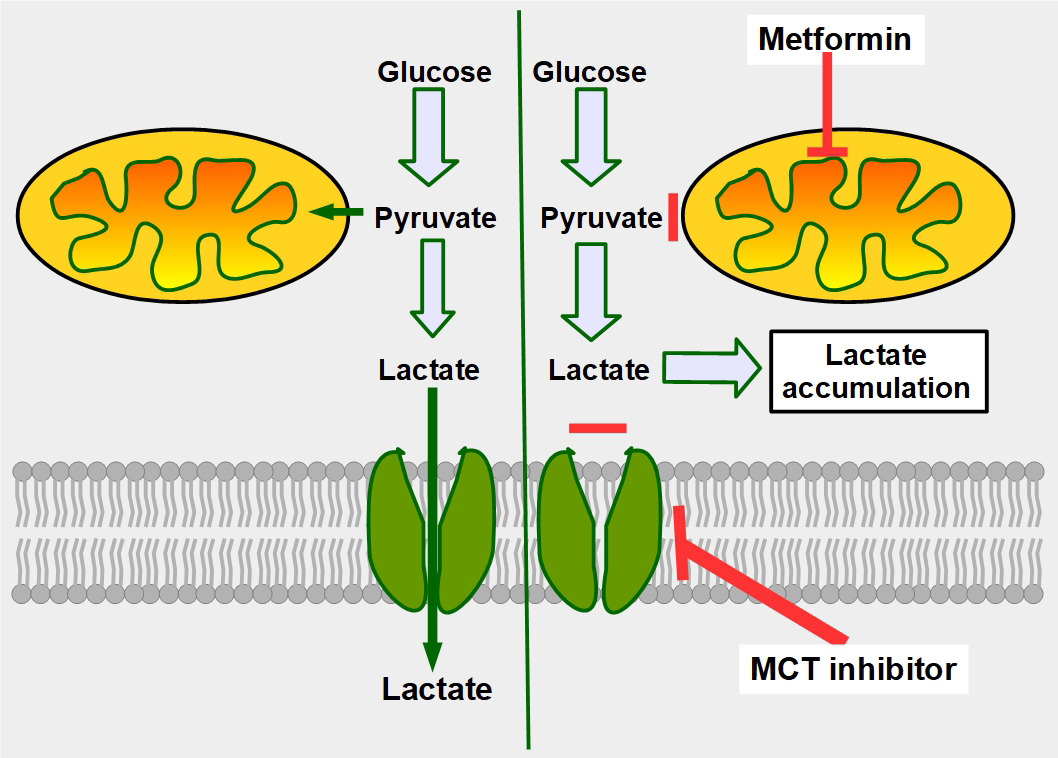
Mechanism of synergy between metformin and MCT inhibitors. The double-edge mechanism of decreasing mitochondrial metabolism by metformin, plus inhibition of lactate export, increases intracellular lactate generating lactic intracellular acidosis. The left panel shows that cancer cells, no matter how glycolytic they are, still metabolize part of the pyruvate through the mitochondrial oxidative metabolism. The right panel shows the combined effects of decreasing oxidative metabolism (upper red line), and at the same time impeding lactate extrusion (lower red line), leading to intracellular lactate accumulation

Most of these possible co-administration suggestions would not increase toxicity and it is even possible that much lower doses would be effective. Just to mention a few of these associations, we can consider metformin (Figure 17), amiloride, topiramate, acetazolamide, bumetanide, diclofenac, lansoprazole, atorvastatin, and among others.

Atorvastatin, through its MCT4 inhibitory ability, should also be evaluated as a useful association with AZD3965. MCT4 inhibition sensitizes cells to ferroptosis, thus a ferroptotic drug such as erastin or artesunate should be studied for the partnership with MCT inhibitors.

We also suggest that AZD3965 or other novel MTC inhibitors may not be stand-alone drugs. But they represent a very important link in a chain of drugs addressed to create a deep intracellular acidification which will eventually induce apoptosis [207–210]. We propose that MCT inhibitors will be more efficient if, at the same time NHE1, membrane carbonic anhydrases, and vacuolar ATPase proton pumps are simultaneously inhibited [211, 212].

As support for this concept, we mention that it was shown earlier that metformin sensitizes glycolytic cells to MCT inhibitors [213, 214]. The mechanism in this case is that metformin increases lactate production and the MCT inhibitor impedes its cellular extrusion resulting in intracellular acidosis. A mechanism such as this was shown to be effective against radio and chemoresistant glycolytic tumor cells [215]. Van der Vreken et al. [216] showed that co-administration of metformin with syrosingopine increased cytotoxicity against multiple myeloma cells *in vitro* and *in vivo*.

Further support for the approach of co-administering compounds in combination with lactate transport inhibitors, is the result of a study that showed that co-administration of MCT inhibitor with cariporide (a powerful NHE1 blocker), had an additive effect on leukemia cell growth inhibition [217]. In this case, intracellular pH was lowered by the combined treatment.

Aside from NHE1, other proteins that affect intracellular pH may be important in modifying MCT activity. Collectively, the transporters, exchangers, and enzymes that regulate intracellular pH are called the pHtome. Carbonic anhydrases are part of these, and they have been shown to cooperate non-enzymatically with MCTs increasing their activity [218–221]. Also, lactate transport by MCT1 was increased 2-fold when MCT1 was expressed together with NBC (the sodium bicarbonate cotransporter, another member of the pHtome) in Xenoupus oocytes [222]. This last experiment shows that the NBC increases intracellular buffering capacity by importing bicarbonate and this permits an increased lactate influx without decreasing intracellular pH. This evidence shows that the pHtome works in a coordinated manner [223].

Who is the coordinator of the pHtome?

The only identified coordinator is intracellular pH. MCTs are components of the pHtome, and the pHtome has many components so it must be targeted in an integral way. It is insufficient to address only MCTs.

If deep intracellular acidification is achieved it could have detrimental effects on tumor cell growth through several different mechanisms. It would slow down glycolysis. Key enzymes for maintaining a high glycolytic flux have an optimum pH of around 8. The optimum pH for glucokinase and hexokinase II was originally thought to be in the low alkaline level. However, it is now known to be more alkaline, in the range of 8.5–8.7 [224] for glucokinase. For other enzymes, their pH optimums are also alkaline, 7.8 for hexokinase I and 8.1 for hexokinase III [225]. In *Sus scrofa* (wild boar), the optimum pH of hexokinase II is 9. Phosphofructokinase 1 is a rate-limiting enzyme of the glycolytic pathway and its pH optimum is around 8 [226]. It is inhibited by low pH and is fully pH-dependent [227]. Another pathway that would be inhibited by deep intracellular acidification is purine and pyrimidine biosynthesis. All the enzymes involved in this pathway have an optimum pH of around 8 [228]. Deep development of therapies combining intracellular acidification with monocarboxylate inhibitors may thus be a very useful method of curtailing tumor cell growth.

This brings us to the approach of using a cocktail of drugs targeting all the pHtome that is required for the efficient blocking of tumor cell proliferation and eventual induction of apoptosis. It is in conjunction with this cocktail where MCT inhibitors have an import role, not as a stand-alone drug.

Although all efforts are currently directed toward the clinical development of AZD3695, we believe that 7-hydroxycoumarin derivatives may be more efficient in a multidrug attack of the pHtome. The reason for this belief is that 7ACC2 can inhibit the mitochondrial pyruvate carrier which sits at the “crossroad of glycolysis” [229]. Limits imposed by its toxicity can perhaps be eliminated through new derivatives.

Finally, it must be mentioned the recent publication by Blaszczak et al. [230] that casts doubt on the utility of MCT inhibition. According to this paper, they showed that MCT inhibition alone cannot reduce lactate production or extrusion because of autoregulatory mechanisms “[compensatory increase in the transmembrane (lactate) driving force]” that would keep a high glycolysis level even in the presence of these inhibitors, and a continuous need to increase their concentration to achieve a result. As these drugs are quite toxic, continuous increase of dose is not a possibility. However, we believe that if MCT inhibitors are complemented with drugs targeting the pHtome, the compensatory increase in the transmembrane driving force would be disrupted.

To the best of our knowledge, lonidamine has never been tested in association with newer MCT inhibitors, such as AZD3695. On a speculative basis, we think it may allow the use of a lower AZD3695 dose and increase its antitumoral effects.

**Figure 18:**
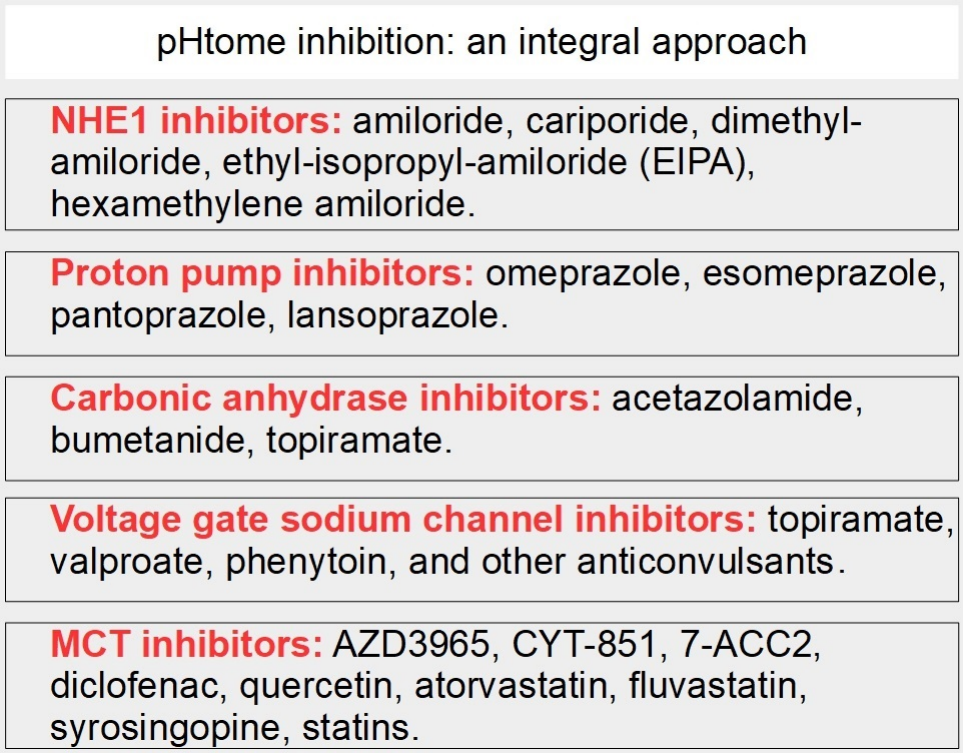
MCT inhibition as part of an integral approach targeting the pHtome

The drugs that can be co-administered with MCT inhibitors to achieve an integral downregulation of proton export are shown in Figure 18 which is based on references [231–237].

## Conclusions

Summarizing the concepts discussed in this paper we can arrive to the following conclusions:

(A) MCTs are valid targets in cancer treatment.
(B) The most potent MCT inhibitor available now at the clinical level (not yet approved) when used alone, is insufficient to make an important difference in cancer treatment.
(C) Effects of AZD3659 should be increased with an integral targeting of the pHtome.
(D) This can be achieved with existing drugs (Figure 18). A combined attack on the entire pHtome is feasible with available drugs.
(E) These associated drugs would not add toxicity to AZD3659, but on the contrary, would allow for a reduction in dose and decrease the toxicity of AZD3659.
(F) It seems insufficient to target MCTs without targeting the other channels, exchangers, and enzymes that regulate intracellular pH.
(G) MCT inhibitors as stand-alone drugs have little chance of being successful unless they are complemented with drugs that can integrally target the altered pH homeostasis of cancer cells.

## Abbreviations

3-D: three-dimensional
7ACC2: 7-aminocarboxycoumarin 2
AMPK: adenosine monophosphate-activated protein kinase
ATPs: adenosine triphosphates
EMMPRIN: extracellular matrix metalloproteinase inducer
MCTs: monocarboxylate transporters
MMPs: matrix metalloproteases
NBC1: sodium bicarbonate cotransporter 1
NHE1: sodium hydrogen exchanger 1
NSAIDs: non-steroidal anti-inflammatory drugs
SLCs: solute carriers

## Declarations

### Copyright

© The Author(s) 2024.

## References

[1] Kroemer G, Pouyssegur J (2008). Tumor cell metabolism: cancer’s Achilles’ heel. Cancer Cell.

[2] Hanahan D, Weinberg RA (2011). Hallmarks of cancer: the next generation. Cell.

[3] Warburg O (1924). Über den stoffwechsel der carcinomzelle. Naturwissenschaften.

[4] Warburg O, Wind F, Negelein E (1927). The metabolism of tumors in the body. J Gen Physiol.

[5] Liu H, Hu YP, Savaraj N, Priebe W, Lampidis TJ (2001). Hypersensitization of tumor cells to glycolytic inhibitors. Biochemistry.

[6] Doherty JR, Yang C, Scott KEN, Cameron MD, Fallahi M, Li W (2014). Blocking lactate export by inhibiting the Myc target MCT1 Disables glycolysis and glutathione synthesis. Cancer Res.

[7] DeBerardinis RJ, Chandel NS (2016). Fundamentals of cancer metabolism. Sci Adv.

[8] Puri S, Juvale K (2020). Monocarboxylate transporter 1 and 4 inhibitors as potential therapeutics for treating solid tumours: a review with structure-activity relationship insights. Eur J Med Chem.

[9] Bosshart PD, Kalbermatter D, Bonetti S, Fotiadis D (2019). Mechanistic basis of L-lactate transport in the SLC16 solute carrier family. Nat Commun.

[10] Halestrap AP, Wilson MC (2012). The monocarboxylate transporter family—role and regulation. IUBMB Life.

[11] Miyauchi S, Gopal E, Fei YJ, Ganapathy V (2004). Functional identification of SLC5A8, a tumor suppressor down-regulated in colon cancer, as a Na^+^-coupled transporter for short-chain fatty acids. J Biol Chem.

[12] Gopal E, Fei YJ, Sugawara M, Miyauchi S, Zhuang L, Martin P (2004). Expression of *slc5a8* in kidney and its role in Na^+^-coupled transport of lactate. J Biol Chem.

[13] Ganapathy V, Gopal E, Miyauchi S, Prasad PD (2005). Biological functions of SLC5A8, a candidate tumour suppressor. Biochem Soc Trans.

[14] Gupta N, Martin PM, Prasad PD, Ganapathy V (2006). SLC5A8 (SMCT1)-mediated transport of butyrate forms the basis for the tumor suppressive function of the transporter. Life Sci.

[15] Nabeshima K, Iwasaki H, Koga K, Hojo H, Suzumiya J, Kikuchi M (2006). Emmprin (basigin/CD147): matrix metalloproteinase modulator and multifunctional cell recognition molecule that plays a critical role in cancer progression. Pathol Int.

[16] Halestrap AP, Price NT (1999). The proton-linked monocarboxylate transporter (MCT) family: structure, function and regulation. Biochem J.

[17] Zhang B, Jin Q, Xu L, Li N, Meng Y, Chang S (2020). Cooperative transport mechanism of human monocarboxylate transporter 2. Nat Commun.

[18] Poole RC, Sansom CE, Halestrap AP (1996). Studies of the membrane topology of the rat erythrocyte H^+^/lactate cotransporter (MCT1). Biochem J.

[19] Poole RC, Halestrap AP (1997). Interaction of the erythrocyte lactate transporter (monocarboxylate transporter 1) with an integral 70-kDa membrane glycoprotein of the immunoglobulin superfamily. J Biol Chem.

[20] Pérez-Escuredo J, Van Hée VF, Sboarina M, Falces J, Payen VL, Pellerin L (2016). Monocarboxylate transporters in the brain and in cancer. Biochim Biophys Acta.

[21] Liao CG, Kong LM, Song F, Xing JL, Wang LX, Sun ZJ (2011). Characterization of basigin isoforms and the inhibitory function of basigin-3 in human hepatocellular carcinoma proliferation and invasion. Mol Cell Biol.

[22] Halestrap AP (2013). The SLC16 gene family – structure, role and regulation in health and disease. Mol Aspects Med.

[23] Kumar D, Vetrivel U, Parameswaran S, Subramanian KK (2019). Structural insights on druggable hotspots in CD147: a bull’s eye view. Life Sci.

[24] Wilson MC, Meredith D, Halestrap AP (2002). Fluorescence resonance energy transfer studies on the interaction between the lactate transporter MCT1 and CD147 provide information on the topology and stoichiometry of the complex *in situ*. J Biol Chem.

[25] Yoshida S, Shibata M, Yamamoto S, Hagihara M, Asai N, Takahashi M (2000). Homo-oligomer formation by basigin, an immunoglobulin superfamily member, via its N-terminal immunoglobulin domain. Eur J Biochem.

[26] Belton RJ Jr, Chen L, Mesquita FS, Nowak RA (2008). Basigin-2 is a cell surface receptor for soluble basigin ligand. J Biol Chem.

[27] Muramatsu T (2016). Basigin (CD147), a multifunctional transmembrane glycoprotein with various binding partners. J Biochem.

[28] Chambers PW (2011). Basigin binds spike S on SARS-CoV-2. OALib Journal.

[29] Muramatsu T (2012). Basigin: a multifunctional membrane protein with an emerging role in infections by malaria parasites. Expert Opin Ther Targets.

[30] Kirk P, Wilson MC, Heddle C, Brown MH, Barclay AN, Halestrap AP (2000). CD147 is tightly associated with lactate transporters MCT1 and MCT4 and facilitates their cell surface expression. EMBO J.

[31] Kanekura T (2023). CD147/basigin is involved in the development of malignant tumors and T-cell-mediated immunological disorders via regulation of glycolysis. Int J Mol Sci.

[32] Kennedy KM, Dewhirst MW (2010). Tumor metabolism of lactate: the influence and therapeutic potential for MCT and CD147 regulation. Future Oncol.

[33] Su J, Chen X, Kanekura T (2008). CD147/basigin plays an important role in tumor glycolysis in association with monocarboxylate transporter. Cancer Res.

[34] Kendrick AA, Schafer J, Dzieciatkowska M, Nemkov T, D’Alessandro A, Neelakantan D (2017). CD147: a small molecule transporter ancillary protein at the crossroad of multiple hallmarks of cancer and metabolic reprogramming. Oncotarget.

[35] Mannowetz N, Wandernoth P, Wennemuth G (2012). Basigin interacts with both MCT1 and MCT2 in murine spermatozoa. J Cell Physiol.

[36] Miyauchi T, Masuzawa Y, Muramatsu T (1991). The basigin group of the immunoglobulin superfamily: complete conservation of a segment in and around transmembrane domains of human and mouse basigin and chicken HT7 antigen. J Biochem.

[37] Biswas C, Zhang Y, DeCastro R, Guo H, Nakamura T, Kataoka H (1995). The human tumor cell-derived collagenase stimulatory factor (renamed EMMPRIN) is a member of the immunoglobulin superfamily. Cancer Res.

[38] Zhou S, Zhou H, Walian PJ, Jap BK (2005). CD147 is a regulatory subunit of the γ-secretase complex in Alzheimer’s disease amyloid β-peptide production. Proc Natl Acad Sci U S A.

[39] Schreiner A, Ruonala M, Jakob V, Suthaus J, Boles E, Wouters F (2007). Junction protein shrew-1 influences cell invasion and interacts with invasion-promoting protein CD147. Mol Biol Cell.

[40] Philp NJ, Ochrietor JD, Rudoy C, Muramatsu T, Linser PJ (2003). Loss of MCT1, MCT3, and MCT4 expression in the retinal pigment epithelium and neural retina of the 5A11/basigin-null mouse. Invest Ophthalmol Vis Sci.

[41] Marchiq I, Le Floch R, Roux D, Simon MP, Pouyssegur J (2015). Genetic disruption of lactate/H^+^ symporters (MCTs) and their subunit CD147/BASIGIN sensitizes glycolytic tumor cells to phenformin. Cancer Res.

[42] Wang N, Jiang X, Zhang S, Zhu A, Yuan Y, Xu H (2021). Structural basis of human monocarboxylate transporter 1 inhibition by anti-cancer drug candidates. Cell.

[43] Brooks GA (2018). The science and translation of lactate shuttle theory. Cell Metab.

[44] Brooks GA, Arevalo JA, Osmond AD, Leija RG, Curl CC, Tovar AP (2022). Lactate in contemporary biology: a phoenix risen. J Physiol.

[45] Brooks GA (2009). Cell-cell and intracellular lactate shuttles. J Physiol.

[46] Hertz L (2004). The astrocyte-neuron lactate shuttle: a challenge of a challenge. J Cereb Blood Flow Metab.

[47] Brooks GA (1986). The lactate shuttle during exercise and recovery. Med Sci Sports Exerc.

[48] Goodwin ML, Gladden LB, Nijsten MW, Jones KB (2015). Lactate and cancer: revisiting the Warburg effect in an era of lactate shuttling. Front Nutr.

[49] Sanità P, Capulli M, Teti A, Galatioto GP, Vicentini C, Chiarugi P (2014). Tumor-stroma metabolic relationship based on lactate shuttle can sustain prostate cancer progression. BMC Cancer.

[50] Bonuccelli G, Tsirigos A, Whitaker-Menezes D, Pavlides S, Pestell RG, Chiavarina B (2010). Ketones and lactate “fuel” tumor growth and metastasis: evidence that epithelial cancer cells use oxidative mitochondrial metabolism. Cell Cycle.

[51] Pavlides S, Whitaker-Menezes D, Castello-Cros R, Flomenberg N, Witkiewicz AK, Frank PG (2009). The reverse Warburg effect: aerobic glycolysis in cancer associated fibroblasts and the tumor stroma. Cell Cycle.

[52] Liang L, Li W, Li X, Jin X, Liao Q, Li Y, Zhou Y (2022). ‘Reverse Warburg effect’ of cancerassociated fibroblasts (review). Int J Oncol.

[53] Manning Fox JE, Meredith D, Halestrap AP (2000). Characterisation of human monocarboxylate transporter 4 substantiates its role in lactic acid efflux from skeletal muscle. J Physiol.

[54] Contreras-Baeza Y, Sandoval PY, Alarcón R, Galaz A, Cortés-Molina F, Alegría K (2019). Monocarboxylate transporter 4 (MCT4) is a high affinity transporter capable of exporting lactate in high-lactate microenvironments. J Biol Chem.

[55] Dimmer KS, Friedrich B, Lang F, Deitmer JW, Bröer S (2000). The low-affinity monocarboxylate transporter MCT4 is adapted to the export of lactate in highly glycolytic cells. Biochem J.

[56] Gatenby RA, Gillies RJ (2007). Glycolysis in cancer: a potential target for therapy. Int J Biochem Cell Biol.

[57] Liberti MV, Locasale JW (2016). The Warburg effect: how does it benefit cancer cells?. Trends Biochem Sci.

[58] DeBerardinis RJ, Chandel NS (2020). We need to talk about the Warburg effect. Nat Metab.

[59] Smallbone K, Gatenby RA, Gillies RJ, Maini PK, Gavaghan DJ (2007). Metabolic changes during carcinogenesis: potential impact on invasiveness. J Theor Biol.

[60] Pinheiro C, Longatto-Filho A, Azevedo-Silva J, Casal M, Schmitt FC, Baltazar F (2012). Role of monocarboxylate transporters in human cancers: state of the art. J Bioenerg Biomembr.

[61] Pinheiro C, Albergaria A, Paredes J, Sousa B, Dufloth R, Vieira D (2010). Monocarboxylate transporter 1 is up-regulated in basal-like breast carcinoma. Histopathology.

[62] Xiao S, Zhu H, Shi Y, Wu Z, Wu H, Xie M (2020). Prognostic and predictive value of monocarboxylate transporter 4 in patients with breast cancer. Oncol Lett.

[63] Yuan C, Zhang J, Lou J, Wang S, Jiang Y, Wu F, Wang S (2021). Comprehensive analysis of monocarboxylate transporter 4 (MCT4) expression in breast cancer prognosis and immune infiltration via integrated bioinformatics analysis. Bioengineered.

[64] Pinheiro C, Longatto-Filho A, Scapulatempo C, Ferreira L, Martins S, Pellerin L (2008). Increased expression of monocarboxylate transporters 1, 2, and 4 in colorectal carcinomas. Virchows Arch.

[65] Gotanda Y, Akagi Y, Kawahara A, Kinugasa T, Yoshida T, Ryu Y (2013). Expression of monocarboxylate transporter (MCT)-4 in colorectal cancer and its role: MCT4 contributes to the growth of colorectal cancer with vascular endothelial growth factor. Anticancer Res.

[66] Abe Y, Nakayama Y, Katsuki T, Inoue Y, Minagawa N, Torigoe T (2019). The prognostic significance of the expression of monocarboxylate transporter 4 in patients with right- or left-sided colorectal cancer. Asia Pac J Clin Oncol.

[67] Pértega-Gomes N, Vizcaíno JR, Miranda-Gonçalves V, Pinheiro C, Silva J, Pereira H (2011). Monocarboxylate transporter 4 (MCT4) and CD147 overexpression is associated with poor prognosis in prostate cancer. BMC Cancer.

[68] Pereira-Nunes A, Simões-Sousa S, Pinheiro C, Miranda-Gonçalves V, Granja S, Baltazar F (2020). Targeting lactate production and efflux in prostate cancer. Biochim Biophys Acta Mol Basis Dis.

[69] Vovdenko S, Morozov A, Ali S, Kogan E, Bezrukov E (2023). Role of monocarboxylate transporters and glucose transporters in prostate cancer. Urologia.

[70] Gao HJ, Zhao MC, Zhang YJ, Zhou DS, Xu L, Li GB (2015). Monocarboxylate transporter 4 predicts poor prognosis in hepatocellular carcinoma and is associated with cell proliferation and migration. J Cancer Res Clin Oncol.

[71] Kuo TC, Huang KY, Yang SC, Wu S, Chung WC, Chang YL (2020). Monocarboxylate transporter 4 is a therapeutic target in non-small cell lung cancer with aerobic glycolysis preference. Mol Ther Oncolytics.

[72] Afonso J, Barbosa A, Aguiar Pastrez PR, Bonatelli M, Alves da Costa RF, Pinheiro C (2023). Clinical and prognostic impact of the Warburg effect in esophageal carcinoma: monocarboxylate transporters as candidates for therapeutic targeting. Pathobiology.

[73] Chen X, Chen X, Liu F, Yuan Q, Zhang K, Zhou W (2019). Monocarboxylate transporter 1 is an independent prognostic factor in esophageal squamous cell carcinoma. Oncol Rep.

[74] Blackhall F (2015). O11.5 - activity of the monocarboxylate transporter 1 inhibitor AZD3965 in small cell lung cancer. Ann Oncol.

[75] Chen H, Wang L, Beretov J, Hao J, Xiao W, Li Y (2010). Co-expression of CD147/EMMPRIN with monocarboxylate transporters and multiple drug resistance proteins is associated with epithelial ovarian cancer progression. Clin Exp Metastasis.

[76] Eskuri M, Kemi N, Kauppila JH (2021). Monocarboxylate transporters 1 and 4 and MTCO1 in gastric cancer. Cancers (Basel).

[77] Yan P, Li YH, Tang ZJ, Shu X, Liu X (2014). High monocarboxylate transporter 4 protein expression in stromal cells predicts adverse survival in gastric cancer. Asian Pac J Cancer Prev.

[78] Payen VL, Mina E, Van Hée VF, Porporato PE, Sonveaux P (2020). Monocarboxylate transporters in cancer. Mol Metab.

[79] Silva A, Cerqueira MC, Rosa B, Sobral C, Pinto-Ribeiro F, Costa MF (2023). Prognostic value of monocarboxylate transporter 1 overexpression in cancer: a systematic review. Int J Mol Sci.

[80] Li M, Long X, Wan H, Yin M, Yang B, Zhang F (2021). Monocarboxylate transporter 1 promotes proliferation and invasion of renal cancer cells by mediating acetate transport. Cell Biol Int.

[81] Guo C, Huang T, Wang QH, Li H, Khanal A, Kang EH (2019). Monocarboxylate transporter 1 and monocarboxylate transporter 4 in cancer-endothelial co-culturing microenvironments promote proliferation, migration, and invasion of renal cancer cells. Cancer Cell Int.

[82] Cao YW, Liu Y, Dong Z, Guo L, Kang EH, Wang YH (2018). Monocarboxylate transporters MCT1 and MCT4 are independent prognostic biomarkers for the survival of patients with clear cell renal cell carcinoma and those receiving therapy targeting angiogenesis. Urol Oncol.

[83] Min X, Cheng H, Cao X, Chen Z, Zhang X, Li Y (2022). Heat shock protein A12A activates migration of hepatocellular carcinoma cells in a monocarboxylate transporter 4-dependent manner. Cell Stress Chaperones.

[84] Fang Y, Liu W, Tang Z, Ji X, Zhou Y, Song S (2023). Monocarboxylate transporter 4 inhibition potentiates hepatocellular carcinoma immunotherapy through enhancing T cell infiltration and immune attack. Hepatology.

[85] Kong SC, Nøhr-Nielsen A, Zeeberg K, Reshkin SJ, Hoffmann EK, Novak I (2016). Monocarboxylate transporters MCT1 and MCT4 regulate migration and invasion of pancreatic ductal adenocarcinoma cells. Pancreas.

[86] Ufuk A, Garner T, Stevens A, Latif A (2022). Monocarboxylate transporters are involved in extracellular matrix remodelling in pancreatic ductal adenocarcinoma. Cancers (Basel).

[87] Wu DH, Liang H, Lu SN, Wang H, Su ZL, Zhang L (2018). miR-124 suppresses pancreatic ductal adenocarcinoma growth by regulating monocarboxylate transporter 1-mediated cancer lactate metabolism. Cell Physiol Biochem.

[88] Lopes-Coelho F, Nunes C, Gouveia-Fernandes S, Rosas R, Silva F, Gameiro P (2017). Monocarboxylate transporter 1 (MCT1), a tool to stratify acute myeloid leukemia (AML) patients and a vehicle to kill cancer cells. Oncotarget.

[89] Saulle E, Spinello I, Quaranta MT, Pasquini L, Pelosi E, Iorio E (2021). Targeting lactate metabolism by inhibiting MCT1 or MCT4 impairs leukemic cell proliferation, induces two different related death-pathways and increases chemotherapeutic sensitivity of acute myeloid leukemia cells. Front Oncol.

[90] Zhao H, Chen Y, Liao YP, Chen HM, Yang QH, Xiao Y (2023). Immunohistochemical evaluation and prognostic value of monocarboxylate transporter 1 (MCT1) and 4 (MCT4) in T-cell non-Hodgkin lymphoma. Clin Exp Med.

[91] Choi JW, Lee Y, Kim H, Cho HY, Min SK, Kim YS (2022). Coexpression of MCT1 and MCT4 in ALK-positive anaplastic large cell lymphoma: diagnostic and therapeutic implications. Am J Surg Pathol.

[92] Noble RA, Bell N, Blair H, Sikka A, Thomas H, Phillips N (2017). Inhibition of monocarboxyate transporter 1 by AZD3965 as a novel therapeutic approach for diffuse large B-cell lymphoma and Burkitt lymphoma. Haematologica.

[93] Chandel V, Maru S, Kumar A, Kumar A, Sharma A, Rathi B (2021). Role of monocarboxylate transporters in head and neck squamous cell carcinoma. Life Sci.

[94] Wang Y, Li Y, Jiang L, Ren X, Cheng B, Xia J (2021). Prognostic value of glycolysis markers in head and neck squamous cell carcinoma: a meta-analysis. Aging (Albany NY).

[95] Chandel V, Kumar D (2021). Targeting signalling cross-talk between cancer cells and cancer-associated fibroblast through monocarboxylate transporters in head and neck cancer. Anticancer Agents Med Chem.

[96] Miranda-Gonçalves V, Honavar M, Pinheiro C, Martinho O, Pires MM, Pinheiro C (2013). Monocarboxylate transporters (MCTs) in gliomas: expression and exploitation as therapeutic targets. Neuro Oncol.

[97] Pinheiro C, Penna V, Morais-Santos F, Abrahão-Machado LF, Ribeiro G, Curcelli EC (2014). Characterization of monocarboxylate transporters (MCTs) expression in soft tissue sarcomas: distinct prognostic impact of MCT1 sub-cellular localization. J Transl Med.

[98] Silva ECA, Cárcano FM, Bonatelli M, Zaia MG, Morais-Santos F, Baltazar F (2018). The clinicopathological significance of monocarboxylate transporters in testicular germ cell tumors. Oncotarget.

[99] Baltazar F, Pinheiro C, Morais-Santos F, Azevedo-Silva J, Queirós O, Preto A (2014). Monocarboxylate transporters as targets and mediators in cancer therapy response. Histol Histopathol.

[100] Jones RS, Morris ME (2016). Monocarboxylate transporters: therapeutic targets and prognostic factors in disease. Clin Pharmacol Ther.

[101] Blaszczak W, Swietach P (2023). Permeability and driving force: why is difficult to control glycolytic flux by blocking lactate transporters?. Oncotarget.

[102] Bovenzi CD, Hamilton J, Tassone P, Johnson J, Cognetti DM, Luginbuhl A (2015). Prognostic indications of elevated MCT4 and CD147 across cancer types: a meta-analysis. Biomed Res Int.

[103] Zhao SH, Wang Y, Wen L, Zhai ZB, Ai ZH, Yao NL (2013). Basigin-2 is the predominant basigin isoform that promotes tumor cell migration and invasion and correlates with poor prognosis in epithelial ovarian cancer. J Transl Med.

[104] Su J, Chen X, Kanekura T (2009). A CD147-targeting siRNA inhibits the proliferation, invasiveness, and VEGF production of human malignant melanoma cells by down-regulating glycolysis. Cancer Lett.

[105] Granja S, Marchiq I, Le Floch R, Moura CS, Baltazar F, Pouysségur J (2015). Disruption of BASIGIN decreases lactic acid export and sensitizes non-small cell lung cancer to biguanides independently of the LKB1 status. Oncotarget.

[106] Cui J, Huang W, Wu B, Jin J, Jing L, Shi WP (2018). *N*-glycosylation by *N*-acetylglucosaminyltransferase V enhances the interaction of CD147/basigin with integrin β1 and promotes HCC metastasis. J Pathol.

[107] Fu TY, Chang CC, Lin CT, Lai CH, Peng SY, Ko YJ (2011). Let-7b-mediated suppression of basigin expression and metastasis in mouse melanoma cells. Exp Cell Res.

[108] Pisarsky L, Bill R, Fagiani E, Dimeloe S, Goosen RW, Hagmann J (2016). Targeting metabolic symbiosis to overcome resistance to anti-angiogenic therapy. Cell Rep.

[109] Koltai T, Fliegel L, Reshkin SJ, Baltazar F, Cardone RA, Alfarouk KO (2023). pH deregulation as the eleventh hallmark of cancer.

[110] Webb BA, Chimenti M, Jacobson MP, Barber DL (2011). Dysregulated pH: a perfect storm for cancer progression. Nat Rev Cancer.

[111] Sonveaux P, Copetti T, De Saedeleer CJ, Végran F, Verrax J, Kennedy KM (2012). Targeting the lactate transporter MCT1 in endothelial cells inhibits lactate-induced HIF-1 activation and tumor angiogenesis. PLoS One.

[112] Schneiderhan W, Scheler M, Holzmann KH, Marx M, Gschwend JE, Bucholz M (2009). CD147 silencing inhibits lactate transport and reduces malignant potential of pancreatic cancer cells in *in vivo* and *in vitro* models. Gut.

[113] Izumi H, Takahashi M, Uramoto H, Nakayama Y, Oyama T, Wang KY (2011). Monocarboxylate transporters 1 and 4 are involved in the invasion activity of human lung cancer cells. Cancer Sci.

[114] Zhao Y, Li M, Yao X, Fei Y, Lin Z, Li Z (2020). HCAR1/MCT1 regulates tumor ferroptosis through the lactate-mediated AMPK-SCD1 activity and its therapeutic implications. Cell Rep.

[115] Dong S, Zheng L, Jiang T (2023). Loss of lactate/proton monocarboxylate transporter 4 induces ferroptosis via the AMPK/ACC pathway and inhibition of autophagy on human bladder cancer 5637 cell line. J Oncol.

[116] Huang HK, Lee SY, Huang SF, Lin YS, Chao SC, Huang SF (2020). Isoorientin decreases cell migration via decreasing functional activity and molecular expression of proton-linked monocarboxylate transporters in human lung cancer cells. Am J Chin Med.

[117] König B, Fischer S, Schlotte S, Wen G, Eder K, Stangl GI (2010). Monocarboxylate transporter 1 and CD147 are up-regulated by natural and synthetic peroxisome proliferator-activated receptor α agonists in livers of rodents and pigs. Mol Nutr Food Res.

[118] Aherne SA, O’Brien NM (2002). Dietary flavonols: chemistry, food content, and metabolism. Nutrition.

[119] Griffith W (1996). Food phenolics: sources, chemistry, effects, applications: by Fereidoon Shahidi and Marian Naczk, Technomic, 1995. $85.00 (ix + 331 pages) ISBN 1 56676 279 0. Trends Food Sci Technol.

[120] Kelly GS (2011). Quercetin. Monograph. Altern Med Rev.

[121] Panche AN, Diwan AD, Chandra SR (2016). Flavonoids: an overview. J Nutr Sci.

[122] Yarahmadi A, Khademi F, Mostafavi-Pour Z, Zal F (2018). *In-vitro* analysis of glucose and quercetin effects on m-TOR and Nrf-2 expression in HepG2 cell line (diabetes and cancer connection). Nutr Cancer.

[123] Badolato M, Carullo G, Perri M, Cione E, Manetti F, Di Gioia ML (2017). Quercetin/oleic acid-based G-protein-coupled receptor 40 ligands as new insulin secretion modulators. Future Med Chem.

[124] Rauf A, Imran M, Khan IA, Ur-Rehman M, Gilani SA, Mehmood Z (2018). Anticancer potential of quercetin: a comprehensive review. Phytother Res.

[125] Ezzati M, Yousefi B, Velaei K, Safa A (2020). A review on anti-cancer properties of Quercetin in breast cancer. Life Sci.

[126] Carpenedo F, Bortignon C, Bruni A, Santi R (1969). Effect of quercetin on membrane-linked activities. Biochem Pharmacol.

[127] Suolinna EM, Lang DR, Racker E (1974). Quercetin, an artificial regulator of the high aerobic glycolysis of tumor cells. J Natl Cancer Inst.

[128] Lang DR, Racker E (1974). Effects of quercetin and F_1_ inhibitor on mitochondrial ATPase and energy-linked reactions in submitochondrial particles. Biochim Biophys Acta.

[129] Suolinna EM, Buchsbaum RN, Racker E (1975). The effect of flavonoids on aerobic glycolysis and growth of tumor cells. Cancer Res.

[130] Belt JA, Thomas JA, Buchsbaum RN, Racker E (1979). Inhibition of lactate transport and glycolysis in Ehrlich ascites tumor cells by bioflavonoids. Biochemistry.

[131] Volk C, Kempski B, Kempski OS (1997). Inhibition of lactate export by quercetin acidifies rat glial cells *in vitro*. Neurosci Lett.

[132] Albatany M, Meakin S, Bartha R (2019). The monocarboxylate transporter inhibitor quercetin induces intracellular acidification in a mouse model of glioblastoma multiforme: *in-vivo* detection using magnetic resonance imaging. Invest New Drugs.

[133] Weigt H, Staub F, Baethmann A, Kempski O, Grote J, Witzleb E (1994). Inhibition of lactate transport causes glial swelling. Funktionsanalyse Biologischer Systeme 22.

[134] Graefe EU, Wittig J, Mueller S, Riethling AK, Uehleke B, Drewelow B (2001). Pharmacokinetics and bioavailability of quercetin glycosides in humans. J Clin Pharmacol.

[135] Riva A, Ronchi M, Petrangolini G, Bosisio S, Allegrini P (2019). Improved oral absorption of quercetin from Quercetin Phytosome^®^, a new delivery system based on food grade lecithin. Eur J Drug Metab Pharmacokinet.

[136] DI Pierro F, Khan A, Bertuccioli A, Maffioli P, Derosa G, Khan S (2021). Quercetin Phytosome^®^ as a potential candidate for managing COVID-19. Minerva Gastroenterol (Torino).

[137] Barras A, Mezzetti A, Richard A, Lazzaroni S, Roux S, Melnyk P (2009). Formulation and characterization of polyphenol-loaded lipid nanocapsules. Int J Pharm.

[138] Vinayak M, Maurya AK (2019). Quercetin loaded nanoparticles in targeting cancer: recent development. Anticancer Agents Med Chem.

[139] Gottfried E, Lang SA, Renner K, Bosserhoff A, Gronwald W, Rehli M (2013). New aspects of an old drug – diclofenac targets MYC and glucose metabolism in tumor cells. PLoS One.

[140] Rodriguez-Cruz V, Ren T, Morris ME (2021). Drug-drug interaction between diclofenac and gamma-hydroxybutyric acid. Biopharm Drug Dispos.

[141] Shintaku K, Hori S, Tsujimoto M, Nagata H, Satoh S, Tsukimori K (2009). Transplacental pharmacokinetics of diclofenac in perfused human placenta. Drug Metab Dispos.

[142] Choi JS, Jin MJ, Han HK (2005). Role of monocarboxylic acid transporters in the cellular uptake of NSAIDs. J Pharm Pharmacol.

[143] Sasaki S, Futagi Y, Ideno M, Kobayashi M, Narumi K, Furugen A (2016). Effect of diclofenac on SLC16A3/MCT4 by the Caco-2 cell line. Drug Metab Pharmacokinet.

[144] Renner K, Bruss C, Schnell A, Koehl G, Becker HM, Fante M (2019). Restricting glycolysis preserves T cell effector functions and augments checkpoint therapy. Cell Rep.

[145] Ananth S, Zhuang L, Gopal E, Itagaki S, Ellappan B, Smith SB (2010). Diclofenac-induced stimulation of SMCT1 (SLC5A8) in a heterologous expression system: a RPE specific phenomenon. Biochem Biophys Res Commun.

[146] Buyse C, Joudiou N, Warscotte A, Richiardone E, Mignion L, Corbet C (2022). Evaluation of syrosingopine, an MCT inhibitor, as potential modulator of tumor metabolism and extracellular acidification. Metabolites.

[147] Benjamin D, Colombi M, Hindupur SK, Betz C, Lane HA, El-Shemerly MY (2016). Syrosingopine sensitizes cancer cells to killing by metformin. Sci Adv.

[148] Janjetovic K, Harhaji-Trajkovic L, Misirkic-Marjanovic M, Vucicevic L, Stevanovic D, Zogovic N (2011). *In vitro* and *in vivo* anti-melanoma action of metformin. Eur J Pharmacol.

[149] Melnik S, Dvornikov D, Müller-Decker K, Depner S, Stannek P, Meister M (2018). Cancer cell specific inhibition of Wnt/β-catenin signaling by forced intracellular acidification. Cell Discov.

[150] Wu S, Xu L, He C, Wang P, Qin J, Guo F (2023). Lactate efflux inhibition by syrosingopine/LOD co-loaded nanozyme for synergetic self-replenishing catalytic cancer therapy and immune microenvironment remodeling. Adv Sci (Weinh).

[151] Benjamin D (2023). An acid test for metformin^†^. J Pathol.

[152] Leung YH, Turgeon J, Michaud V (2017). Study of statin- and loratadine-induced muscle pain mechanisms using human skeletal muscle cells. Pharmaceutics.

[153] Leung YH, Lu J, Papillon MÈ, Bélanger F, Turgeon J, Michaud V (2013). The role of MCT1 and MCT4 in drug‐induced muscle disorders. FASEB J.

[154] Kobayashi M, Otsuka Y, Itagaki S, Hirano T, Iseki K (2006). Inhibitory effects of statins on human monocarboxylate transporter 4. Int J Pharm.

[155] Yamaguchi A, Mukai Y, Sakuma T, Furugen A, Narumi K, Kobayashi M (2023). Atorvastatin exerts more selective inhibitory effects on hMCT2 than on hMCT1 and hMCT4. Anticancer Res.

[156] Lin RY, Vera JC, Chaganti RS, Golde DW (1998). Human monocarboxylate transporter 2 (MCT2) is a high affinity pyruvate transporter. J Biol Chem.

[157] du Souich P, Roederer G, Dufour R (2017). Myotoxicity of statins: mechanism of action. Pharmacol Ther.

[158] Kikutani Y, Kobayashi M, Konishi T, Sasaki S, Narumi K, Furugen A (2016). Involvement of monocarboxylate transporter 4 expression in statin-induced cytotoxicity. J Pharm Sci.

[159] Sasaki S, Futagi Y, Ideno M, Kobayashi M, Narumi K, Furugen A (2016). Interaction of atorvastatin with the human glial transporter SLC16A1. Eur J Pharmacol.

[160] Chou YC, Wang YK, Charng MJ, Ueng YF (2013). Determination of serum atorvastatin concentrations in lipid-controlling patients with and without myalgia syndrome. J Food Drug Anal.

[161] Chen W, Tan Q, Guo M, Liao T, Li Y, Yin Z (2023). Tumor cell-derived microparticles packaging monocarboxylate transporter4 inhibitor fluvastatin suppress lung adenocarcinoma via tumor microenvironment remodeling and improve chemotherapy. Chem Eng J.

[162] Floridi A, Paggi MG, Marcante ML, Silvestrini B, Caputo A, De Martino C (1981). Lonidamine, a selective inhibitor of aerobic glycolysis of murine tumor cells. J Natl Cancer Inst.

[163] Caputo A, Silvestrini B (1984). Lonidamine, a new approach to cancer therapy. Oncology.

[164] Nancolas B, Guo L, Zhou R, Nath K, Nelson DS, Leeper DB (2016). The anti-tumour agent lonidamine is a potent inhibitor of the mitochondrial pyruvate carrier and plasma membrane monocarboxylate transporters. Biochem J.

[165] Fang J, Quinones QJ, Holman TL, Morowitz MJ, Wang Q, Zhao H (2006). The H^+^-linked monocarboxylate transporter (MCT1/*SLC16A1*): a potential therapeutic target for high-risk neuroblastoma. Mol Pharmacol.

[166] Nath K, Guo L, Nancolas B, Nelson DS, Shestov AA, Lee SC (2016). Mechanism of antineoplastic activity of lonidamine. Biochim Biophys Acta.

[167] Floridi A, Paggi MG, D’Atri S, De Martino C, Marcante ML, Silvestrini B, Caputo A (1981). Effect of lonidamine on the energy metabolism of Ehrlich ascites tumor cells. Cancer Res.

[168] Paggi MG, Fanciulli M, Perrotti N, Floridi A, Zeuli M, Silvestrini B (1988). The role of mitochondrial hexokinase in neoplastic phenotype and its sensitivity to lonidamine. Ann N Y Acad Sci.

[169] Mathupala SP, Ko YH, Pedersen PL (2009). Hexokinase-2 bound to mitochondria: cancer’s stygian link to the “Warburg effect” and a pivotal target for effective therapy. Semin Cancer Biol.

[170] Belzacq AS, El Hamel C, Vieira HL, Cohen I, Haouzi D, Métivier D (2001). Adenine nucleotide translocator mediates the mitochondrial membrane permeabilization induced by lonidamine, arsenite and CD437. Oncogene.

[171] Ravagnan L, Marzo I, Costantini P, Susin SA, Zamzami N, Petit PX (1999). Lonidamine triggers apoptosis via a direct, Bcl-2-inhibited effect on the mitochondrial permeability transition pore. Oncogene.

[172] Bhutia YD, Babu E, Ganapathy V (2016). Re-programming tumour cell metabolism to treat cancer: no lone target for lonidamine. Biochem J.

[173] Ben-Yoseph O, Lyons JC, Song CW, Ross BD (1998). Mechanism of action of lonidamine in the 9L brain tumor model involves inhibition of lactate efflux and intracellular acidification. J Neurooncol.

[174] Nath K, Nelson DS, Heitjan DF, Zhou R, Leeper DB, Glickson JD (2015). Effects of hyperglycemia on lonidamine-induced acidification and de-energization of human melanoma xenografts and sensitization to melphalan. NMR Biomed.

[175] Xie QR, Liu Y, Shao J, Yang J, Liu T, Zhang T (2013). Male contraceptive Adjudin is a potential anti-cancer drug. Biochem Pharmacol.

[176] Huang Y, Sun G, Sun X, Li F, Zhao L, Zhong R (2020). The potential of lonidamine in combination with chemotherapy and physical therapy in cancer treatment. Cancers (Basel).

[177] Nosova YN, Foteeva LS, Zenin IV, Fetisov TI, Kirsanov KI, Yakubovskaya MG (2017). Enhancing the cytotoxic activity of anticancer Pt^IV^ complexes by introduction of lonidamine as an axial ligand. Eur J Inorg Chem.

[178] Guo W, Kuang Y, Wu J, Wen D, Zhou A, Liao Y (2020). Hexokinase 2 depletion confers sensitization to metformin and inhibits glycolysis in lung squamous cell carcinoma. Front Oncol.

[179] Gatzemeier U, Toomes H, Picollo R, Christoffel V, Lücker PW, Ulmer J (1991). Single- and multiple dose pharmacokinetics of lonidamine in patients suffering from non-small-cell lung cancer. Arzneimittelforschung.

[180] Newell DR, Mansi J, Hardy J, Button D, Jenns K, Smith IE (1991). The pharmacokinetics of oral lonidamine in breast and lung cancer patients. Semin Oncol.

[181] Young CW, Currie VE, Kim JH, O’Hehir MA, Farag FM, Kinahan JE (1984). Phase I and clinical pharmacologic evaluation of Lonidamine in patients with advanced cancer. Oncology.

[182] Jonnalagadda S, Jonnalagadda SK, Ronayne CT, Nelson GL, Solano LN, Rumbley J (2019). Novel N,N-dialkyl cyanocinnamic acids as monocarboxylate transporter 1 and 4 inhibitors. Oncotarget.

[183] Bröer S, Schneider HP, Bröer A, Rahman B, Hamprecht B, Deitmer JW (1998). Characterization of the monocarboxylate transporter 1 expressed in Xenopus laevis oocytes by changes in cytosolic pH. Biochem J.

[184] Nelson GL, Ronayne CT, Solano LN, Jonnalagadda SK, Jonnalagadda S, Rumbley J (2019). Development of novel silyl cyanocinnamic acid derivatives as metabolic plasticity inhibitors for cancer treatment. Sci Rep.

[185] Quanz M, Bender E, Kopitz C, Grünewald S, Schlicker A, Schwede W (2018). Preclinical efficacy of the novel monocarboxylate transporter 1 inhibitor BAY-8002 and associated markers of resistance. Mol Cancer Ther.

[186] Draoui N, Schicke O, Seront E, Bouzin C, Sonveaux P, Riant O (2014). Antitumor activity of 7-aminocarboxycoumarin derivatives, a new class of potent inhibitors of lactate influx but not efflux. Mol Cancer Ther.

[187] Corbet C, Bastien E, Draoui N, Doix B, Mignion L, Jordan BF (2018). Interruption of lactate uptake by inhibiting mitochondrial pyruvate transport unravels direct antitumor and radiosensitizing effects. Nat Commun.

[188] Sandforth L, Ammar N, Dinges LA, Röcken C, Arlt A, Sebens S (2020). Impact of the *monocarboxylate transporter-1* (MCT1)-mediated cellular import of lactate on stemness properties of human pancreatic adenocarcinoma cells. Cancers (Basel).

[189] Park SJ, Smith CP, Wilbur RR, Cain CP, Kallu SR, Valasapalli S (2018). An overview of MCT1 and MCT4 in GBM: small molecule transporters with large implications. Am J Cancer Res.

[190] Halford SE, Jones P, Wedge S, Hirschberg S, Katugampola S, Veal G (2017). A first-in-human first-in-class (FIC) trial of the monocarboxylate transporter 1 (MCT1) inhibitor AZD3965 in patients with advanced solid tumours. J Clin Oncol.

[191] Halford SRE, Walter H, McKay P, Townsend W, Linton K, Heinzmann K (2021). Phase I expansion study of the first-in-class monocarboxylate transporter 1 (MCT1) inhibitor AZD3965 in patients with diffuse large B-cell lymphoma (DLBCL) and Burkitt lymphoma (BL). J Clin Oncol.

[192] Halford S, Veal GJ, Wedge SR, Payne GS, Bacon CM (2023). A phase I dose-escalation study of AZD3965, an oral monocarboxylate transporter 1 inhibitor, in patients with advanced cancer. Clin Cancer Res.

[193] Polański R, Hodgkinson CL, Fusi A, Nonaka D, Priest L, Kelly P (2014). Activity of the monocarboxylate transporter 1 inhibitor AZD3965 in small cell lung cancer. Clin Cancer Res.

[194] Le Floch R, Chiche J, Marchiq I, Naiken T, Ilc K, Murray CM (2011). CD147 subunit of lactate/H^+^ symporters MCT1 and hypoxia-inducible MCT4 is critical for energetics and growth of glycolytic tumors. Proc Natl Acad Sci U S A.

[195] Curtis NJ, Mooney L, Hopcroft L, Michopoulos F, Whalley N, Zhong H (2017). Pre-clinical pharmacology of AZD3965, a selective inhibitor of MCT1: DLBCL, NHL and Burkitt’s lymphoma anti-tumor activity. Oncotarget.

[196] Mehibel M, Ortiz-Martinez F, Voelxen N, Boyers A, Chadwick A, Telfer BA (2018). Statin-induced metabolic reprogramming in head and neck cancer: a biomarker for targeting monocarboxylate transporters. Sci Rep.

[197] Koltai T (2016). Cancer: fundamentals behind pH targeting and the double-edged approach. Onco Targets Ther.

[198] Popović DJ, Popović KJ, Miljković D, Popović JK, Lalošević D, Poša M (2023). Diclofenac and metformin synergistic dose dependent inhibition of hamster fibrosarcoma, rescued with mebendazole. Biomed Pharmacother.

[199] Benjamin D, Hall MN (2022). Combining metformin with lactate transport inhibitors as a treatment modality for cancer - recommendation proposal. Front Oncol.

[200] Lynch RC, Munster PN, Falchook GS, Burness ML, Yap TA, Shapiro G (2023). Phase 1 results of CYT-0851, a monocarboxylate transporter (MCT) inhibitor, in combination with capecitabine (cape) or gemcitabine (gem) in advanced solid tumors. J Clin Oncol.

[201] (2021). Preliminary activity seen with RAD51 inhibitor. Cancer Discov.

[202] Liang Q, Xiong H, Gao G, Xiong K, Wang X, Zhao Z (2005). Inhibition of basigin expression in glioblastoma cell line via antisense RNA reduces tumor cell invasion and angiogenesis. Cancer Biol Ther.

[203] Wilson MC, Meredith D, Fox JEM, Manoharan C, Davies AJ, Halestrap AP (2005). Basigin (CD147) is the target for organomercurial inhibition of monocarboxylate transporter isoforms 1 and 4: the ancillary protein for the insensitive MCT2 is EMBIGIN (gp70). J Biol Chem.

[204] Voss DM, Spina R, Carter DL, Lim KS, Jeffery CJ, Bar EE (2017). Disruption of the monocarboxylate transporter-4-basigin interaction inhibits the hypoxic response, proliferation, and tumor progression. Sci Rep.

[205] Piorecka K, Kurjata J, Stanczyk WA (2022). Acriflavine, an acridine derivative for biomedical application: current state of the art. J Med Chem.

[206] Grasa L, Chueca E, Arechavaleta S, García-González MA, Sáenz MÁ, Valero A (2023). Antitumor effects of lactate transport inhibition on esophageal adenocarcinoma cells. J Physiol Biochem.

[207] Harguindey S, Stanciu D, Devesa J, Alfarouk K, Cardone RA, Polo Orozco JD (2017). Cellular acidification as a new approach to cancer treatment and to the understanding and therapeutics of neurodegenerative diseases. Semin Cancer Biol.

[208] Harguindey S, Alfarouk K, Orozco JP, Hardonniere K, Stanciu D, Fais S (2020). A new and integral approach to the etiopathogenesis and treatment of breast cancer based upon its hydrogen ion dynamics. Int J Mol Sci.

[209] Koltai T (2020). The Ph paradigm in cancer. Eur J Clin Nutr.

[210] Amith SR, Wilkinson JM, Baksh S, Fliegel L (2015). The Na⁺/H⁺ exchanger (NHE1) as a novel co-adjuvant target in paclitaxel therapy of triple-negative breast cancer cells. Oncotarget.

[211] Koltai T, Reshkin SJ, Harguindey S (2020). An innovative approach to understanding and treating cancer: targeting ph: from etiopathogenesis to new therapeutic avenues.

[212] Koltai T (2020). Targeting the pH paradigm at the bedside: a practical approach. Int J Mol Sci.

[213] Floch RL, Chiche J, Marchiq I, Naiken T, Ilc K, Simon MP (2012). Abstract 3225: growth inhibition of glycolytic tumors by targeting basigin/lactate-H+ symporters (MCTs): metformin sensitizes MCT inhibition. Cancer Res.

[214] Benjamin D, Robay D, Hindupur SK, Pohlmann J, Colombi M, El-Shemerly MY (2018). Dual inhibition of the lactate transporters MCT1 and MCT4 is synthetic lethal with metformin due to NAD+ depletion in cancer cells. Cell Rep.

[215] Lee ZW, Teo XY, Song ZJ, Nin DS, Novera W, Choo BA (2017). Intracellular hyper-acidification potentiated by hydrogen sulfide mediates invasive and therapy resistant cancer cell death. Front Pharmacol.

[216] Van der Vreken A, Oudaert I, Ates G, Faict S, Vlummens P, Satilmis H (2023). Metformin confers sensitisation to syrosingopine in multiple myeloma cells by metabolic blockage and inhibition of protein synthesis. J Pathol.

[217] Pivovarova AI, MacGregor GG (2018). Glucose-dependent growth arrest of leukemia cells by MCT1 inhibition: feeding Warburg’s sweet tooth and blocking acid export as an anticancer strategy. Biomed Pharmacother.

[218] Klier M, Andes FT, Deitmer JW, Becker HM (2014). Intracellular and extracellular carbonic anhydrases cooperate non-enzymatically to enhance activity of monocarboxylate transporters. J Biol Chem.

[219] Noor SI, Jamali S, Ames S, Langer S, Deitmer JW, Becker HM (2018). A surface proton antenna in carbonic anhydrase II supports lactate transport in cancer cells. Elife.

[220] Ames S, Pastorekova S, Becker HM (2018). The proteoglycan-like domain of carbonic anhydrase IX mediates non-catalytic facilitation of lactate transport in cancer cells. Oncotarget.

[221] Aspatwar A, Tolvanen MEE, Schneider HP, Becker HM, Narkilahti S, Parkkila S (2019). Catalytically inactive carbonic anhydrase-related proteins enhance transport of lactate by MCT1. FEBS Open Bio.

[222] Becker HM, Bröer S, Deitmer JW (2004). Facilitated lactate transport by MCT1 when coexpressed with the sodium bicarbonate cotransporter (NBC) in Xenopus oocytes. Biophys J.

[223] Granja S, Tavares-Valente D, Queirós O, Baltazar F (2017). Value of pH regulators in the diagnosis, prognosis and treatment of cancer. Semin Cancer Biol.

[224] Šimčíková D, Heneberg P (2019). Identification of alkaline pH optimum of human glucokinase because of ATP-mediated bias correction in outcomes of enzyme assays. Sci Rep.

[225] Sequence of HXK2_HUMAN [Internet]. https://www.brenda-enzymes.org/sequences.php?AC=P52789.

[226] Paetkau V, Lardy HA (1967). Phosphofructokinase. Correlation of physical and enzymatic properties. J Biol Chem.

[227] Bock PE, Frieden C (1976). Phosphofructokinase. I. Mechanism of the pH-dependent inactivation and reactivation of the rabbit muscle enzyme. J Biol Chem.

[228] Alqahtani SS, Koltai T, Ibrahim ME, Bashir AHH, Alhoufie STS, Ahmed SBM (2022). Role of pH in rgulating cncer primidine snthesis. J Xenobiot.

[229] Liu KX, Everdell E, Pal S, Haas-Kogan DA, Milligan MG (2021). Harnessing lactate metabolism for radiosensitization. Front Oncol.

[230] Blaszczak W, Williams H, Swietach P (2022). Autoregulation of H^+^/lactate efflux prevents monocarboxylate transport (MCT) inhibitors from reducing glycolytic lactic acid production. Br J Cancer.

[231] Mihaila RG (2015). A minireview on NHE1 inhibitors. A rediscovered hope in oncohematology. Biomed Pap Med Fac Univ Palacky Olomouc Czech Repub.

[232] Malebari AM, Ibrahim TS, Salem IM, Salama I, Khayyat AN, Mostafa SM (2020). The anticancer activity for the bumetanide-based analogs via targeting the tumor-associated membrane-bound human carbonic anhydrase-IX enzyme. Pharmaceuticals (Basel).

[233] Carta F, Supuran CT (2013). Diuretics with carbonic anhydrase inhibitory action: a patent and literature review (2005 – 2013). Expert Opin Ther Pat.

[234] Fais S (2015). Evidence-based support for the use of proton pump inhibitors in cancer therapy. J Transl Med.

[235] Spugnini E, Fais S (2017). Proton pump inhibition and cancer therapeutics: a specific tumor targeting or it is a phenomenon secondary to a systemic buffering?. Semin Cancer Biol.

[236] Yang M, Kozminski DJ, Wold LA, Modak R, Calhoun JD, Isom LL (2012). Therapeutic potential for phenytoin: targeting Na_v_1.5 sodium channels to reduce migration and invasion in metastatic breast cancer. Breast Cancer Res Treat.

[237] Nelson M, Yang M, Dowle AA, Thomas JR, Brackenbury WJ (2015). The sodium channel-blocking antiepileptic drug phenytoin inhibits breast tumour growth and metastasis. Mol Cancer.

